# The Heisenberg-RIXS instrument at the European XFEL

**DOI:** 10.1107/S1600577524010890

**Published:** 2025-01-01

**Authors:** Justine Schlappa, Giacomo Ghiringhelli, Benjamin E. Van Kuiken, Martin Teichmann, Piter S. Miedema, Jan Torben Delitz, Natalia Gerasimova, Serguei Molodtsov, Luigi Adriano, Bernard Baranasic, Carsten Broers, Robert Carley, Patrick Gessler, Nahid Ghodrati, David Hickin, Le Phuong Hoang, Manuel Izquierdo, Laurent Mercadier, Giuseppe Mercurio, Sergii Parchenko, Marijan Stupar, Zhong Yin, Leonardo Martinelli, Giacomo Merzoni, Ying Ying Peng, Torben Reuss, Sreeju Sreekantan Nair Lalithambika, Simone Techert, Tim Laarmann, Simo Huotari, Christian Schroeter, Burkhard Langer, Tatjana Giessel, Jana Buchheim, Grzegorz Gwalt, Andrey Sokolov, Frank Siewert, Robby Buechner, Vinicius Vaz da Cruz, Sebastian Eckert, Chun-Yu Liu, Christian Sohrt, Christian Weniger, Annette Pietzsch, Stefan Neppl, Friedmar Senf, Andreas Scherz, Alexander Föhlisch

**Affiliations:** a European XFEL Holzkoppel 4 Schenefeld22869 Germany; b Dipartimento di Fisica Politecnico di Milano Piazza Leonardo da Vinci 32 I-20133Milano Italy; c CNR-SPIN, Dipartimento di Fisica Politecnico di Milano I-20133Milano Italy; d Institute of Experimental Physics TU Bergakademie Freiberg Leipziger Str. 23 09599Freiberg Germany; e Center for Efficient High Temperature Processes and Materials Conversion (ZeHS) TU Bergakademie Freiberg Winklerstrasse 5 09599Freiberg Germany; f Deutsches Elektronen-Synchrotron DESY Notkestraße 85 22607Hamburg Germany; g Institute of X-ray Physics, Göttingen University, Friedrich Hund Platz 1, 37077Göttingen, Germany; h The Hamburg Centre for Ultrafast Imaging CUI, Luruper Chaussee 149, 22761Hamburg, Germany; i Department of Physics University of Helsinki PO Box 64 FI-00014Helsinki Finland; j BESTEC GmbH, Am Studio 2b, 12489Berlin, Germany; k Department Optics and Beamlines, Helmholtz-Zentrum Berlin für Materialien und Energie GmbH, Albert-Einstein-Straße 15, 12489Berlin, Germany; l Institute Methods and Instrumentation for Synchrotron Radiation Research Helmholtz-Zentrum Berlin für Materialien und Energie GmbH Albert-Einstein-Straße 15 12489Berlin Germany; m University of Potsdam Institute of Physics and Astronomy Karl-Liebknecht-Straße 24/25 14476Potsdam Germany; RIKEN SPring-8 Center, Japan

**Keywords:** Heisenberg RIXS, hRIXS, resonant inelastic X-ray scattering, RIXS, European XFEL, X-ray Raman scattering, free-electron lasers, soft X-rays, time-resolved spectroscopy, spin dynamics, photochemistry, resonant X-ray diffraction, X-ray resonant diffraction, charge order, spin order

## Abstract

Resonant inelastic X-ray scattering (RIXS) is an ideal X-ray spectroscopy method to push the combination of energy and time resolutions to the Fourier transform ultimate limit, because it is unaffected by the core-hole lifetime energy broadening. Also, in pump–probe experiments the interaction time is made very short by the same core-hole lifetime. RIXS is very photon hungry so it takes great advantage from high-repetition-rate pulsed X-ray sources like the European XFEL. The Heisenberg RIXS instrument is designed for RIXS experiments in the soft X-ray range with energy resolution approaching the Fourier and the Heisenberg limits. It is based on a spherical grating with variable line spacing and a position-sensitive 2D detector. Initially, two gratings were installed to adequately cover the whole photon energy range. With optimized spot size on the sample and small pixel detector the energy resolution can be better than 40 meV (90 meV) at any photon energy below 1000 eV with the high-resolution (high-transmission) grating. At the SCS instrument of the European XFEL the spectrometer can be easily positioned thanks to air pads on a high-quality floor, allowing the scattering angle to be continuously adjusted over the 65–145° range. It can be coupled to two different sample interaction chambers, one for liquid jets and one for solids, each state-of-the-art equipped and compatible for optical laser pumping in collinear geometry. The measured performances, in terms of energy resolution and count rate on the detector, closely match design expectations. The Heisenberg RIXS instrument has been open to public users since the summer of 2022.

## Abstract 2

The aim of this work is to present Heisenberg RIXS at the European XFEL, the first high-resolution soft X-ray instrument enabling time-resolved resonant inelastic X-ray scattering close to the transform limit from solid and liquid-jet samples. The optical design, mechanical layout and performance are described in detail and discussed in a broader context of X-ray spectroscopy at high-repetition free-electron lasers.

## 1. Introduction

At the core of the quantum mechanical description of matter stands uncertainty relations as identified by Heisenberg in 1927 (Heisenberg, 1927 ▸). The position–momentum uncertainty results from the commutation of the position and momentum operators linked to the Planck constant, ℏ. In contrast, the time–energy uncertainty, δ*E*δ*t* ≥ ℏ, does not stand on such fundamental footing, reflecting the fact that time is a variable. How to conceptualize time and time–energy uncertainty relations has been debated over many years (Heisenberg, 1927 ▸; Mandelstam & Tamm, 1945 ▸; Aharonov & Bohm, 1961 ▸) and has led to numerous reviews (Busch, 2008 ▸; Hilgevoord, 2005 ▸). The value of ℏ = 0.66 eV fs makes the time–energy uncertainty central for physics, chemistry, biology and materials science. It relates the femtosecond (fs) timescale of reaction dynamics and materials functionality to the electronvolt (eV) energy scale of valence electrons, chemical bonds as well as spin and magnetic properties.

Unlike for an isolated quantum-mechanical system, the lower limit for the uncertainty relation during a (disturbing) measurement process is given as δ*E*δ*t* ≥ 2πℏ (Messiah, 1999 ▸). The trade-off between energy and time has important implications for spectroscopic and time-resolved techniques in the study of matter. In particular, a parameter that is hard to access in the energy (frequency) domain can be more easily determined in the time domain and vice versa. Time-resolved spectroscopy has thus been rapidly developing in the optical regime thanks to ultra-short laser pulses, which are used both to initially bring the system out of equilibrium (pump) and to observe the transient modifications of its properties (probe) – encoded in the complex optical constants. More recently, the pump–probe scheme has been extended to photoelectron spectroscopy, X-ray absorption and X-ray scattering, which provide direct insights into the electronic structure and ordering phenomena. The idea is that (by refining both the pump and the probe) it will become possible to recognize links among the various microscopic (lattice, charge, orbital and spin) degrees of freedom by selectively exciting one of them and observing changes induced in the other ones.

The advent of X-ray free-electron lasers (XFELs) kicked off the age of selective pump–probe experiments based on X-ray photons. Initially, the inherent fluctuations of intensity and energy in the self-amplified spontaneous emission (SASE) process that creates ultrashort X-ray laser pulses implied that the power density could easily exceed the radiation damage threshold for a majority of materials. This fact effectively limited the average flux actually usable, making photon-hungry techniques challenging at low-repetition-rate XFELs. That is the reason why inelastic X-ray scattering in the time-resolved mode has taken off more slowly than techniques with higher signal at the detector, *i.e.* diffraction, coherent scattering and X-ray absorption spectroscopy. These limitations are now overcome thanks to high-repetition-rate XFELs based on superconducting linac technology, where the energy per X-ray pulse can be adjusted to avoid damaging the sample while preserving the number of photons hitting the sample per second at the level of storage-ring-based experiments, or even higher. RIXS appeared to be the best technique to perform high-quality time-resolved spectroscopy at high-repetition-rate XFELs for a number of reasons. Firstly, it does not involve charged particles (*i.e.* electrons) that are affected by space charge. Secondly, time-resolved RIXS is sensitive to all low-energy excitations of charge, spin, orbital polarization as well as structural distortions and their ultrafast dynamics (Ament *et al.*, 2011 ▸; Gel’mukhanov *et al.*, 2021 ▸). Therefore, RIXS is the ideal spectroscopic tool to advance the science of how to govern materials properties, control chemical and biological processes, or create novel and transient phases that cannot be reached in equilibrium. In particular, since RIXS is a resonant Raman scattering process in the X-ray regime, both Stokes and anti-Stokes features can be used. The latter being univocal markers of the excited states, they can be used to characterize the transient states and their dynamics with superior elemental and chemical selectivity and stringent symmetry selection rules. As a photon-in/photon-out technique, RIXS can access all aggregate states in equilibrium and non-equilibrium. Strong external stimuli such as laser pulses and electromagnetic fields allow for state preparation but do not affect the measurement. Beyond that, the full potential of novel non-linear processes (*e.g.* four-wave mixing with X-rays) can be explored.

The choice of RIXS entails considering another time scale that is defined by the intrinsic lifetime of the intermediate state involved in the second-order resonant scattering process. The Heisenberg relation holds here too, but with little impact on the ultimate time resolution of the pump–probe RIXS experiment, because RIXS usually involves core holes with lifetimes of a few femtoseconds at most, much shorter than the experimental pump–probe resolution. The core-hole lifetime determines the apparent duration of the resonant scattering process, because, within the intrinsic energy width of intermediate states, multi-path interference and energy-detuning effects are significant. These crucial mechanisms are well captured by the Kramers–Heisenberg equation (Gel’mukhanov *et al.*, 2021 ▸). The intermediate state lifetime is a different time scale, independent of the transient state prepared by the pump pulse. As long as the latter are much longer than the former, the RIXS process can be regarded as instantaneous. Conversely, if and when the experimental resolution reaches the femtosecond range and the inelastic X-ray scattering is used to study truly ultrafast dynamics, some precautions will be needed to avoid misinterpretation of the experimental results (Hilgevoord, 1996 ▸; Hilgevoord, 1998 ▸).

At the European X-ray Free Electron Facility GmbH (European XFEL), which provides unprecedented ultra-short soft X-ray pulses with high repetition rate and brightness, the conceptual aspects outlined above can be put into experimental reality and we can finally push time-resolved X-ray spectroscopy closer to the limits of retrievable information. We call this approach Heisenberg RIXS (hRIXS). In this article, we describe the hRIXS spectrometer for soft X-ray photons (200–2000 eV), which is coupled with two experimental environments, one for solid (named the XRD chamber) and one for liquids samples (the CHEM chamber).

The hRIXS approach, proposed by a user consortium and endorsed by the European XFEL management but largely supported by external funding, led to the design and construction of a high-resolution soft X-ray spectrometer to be installed at the SCS instrument. Below we describe the technical design of the hRIXS spectrometer and its performance space. We discuss the underlying criteria and constraints, the final optical design and the actual realization, including the mechanical and detection aspect. The target performances were quickly reached, as confirmed in the commissioning runs of 2021 and 2022. An outlook to all operational parameters is given at the end of the article.

## 2. Requirements and goals of a RIXS experimental setup operating at an X-ray FEL

### 2.1. Soft X-ray RIXS spectroscopy

RIXS is an energy-loss spectroscopy performed with X-ray photons and with their energy tuned to the binding energy of a core level of one of the atomic species present in the material. The resonance greatly enhances the scattering cross section and provides chemical and site selectivity. Moreover, the spin–orbit interaction in the intermediate state with a core hole is often large enough (>5 eV) to trigger pure spin-flip transitions, allowing the study of magnons with RIXS (Braicovich *et al.*, 2010*a* ▸; Ament *et al.*, 2011 ▸). In contrast to inelastic neutron scattering (INS), RIXS also enables observation of non-spin-flip magnetic excitations (Schlappa *et al.*, 2018 ▸). The sizable momentum of X-rays allows the study of collective excitations or quasi-particles in both the energy and momentum domain (already in the soft X-ray range). The potential of RIXS has emerged in the last 15 years, after the experimental bandwidth was improved enough to resolve the physically relevant local and collective excitations in the samples. The bandwidth limit to resolve these excitations is around 100–120 meV, although 40 meV is a standard value in the best facilities and 20 meV is technically feasible in some cases. The task is technically challenging, because the RIXS experiment requires very high resolving power both in the monochromator preparing the beam before the sample and in the spectrometer analysing the scattered radiation: 100 meV bandwidth at 1000 eV photon energy requires 15000 resolving power on each instrument. The low efficiency of the scattering process and the high resolving power imply that a brilliant undulator source, optimized beamline optics to monochromatize the beam and refocus it down to a few-micrometres spot size on the sample, and an efficient spectrometer are all needed to measure high-resolution RIXS spectra.

The reason for choosing the soft X-ray range for high-resolution RIXS is motivated by the presence of the *K* absorption edges of light elements such as C, N and O, the *L*_2,3_ edges of 3*d* transition metals and the *M*_4,5_ edges of 4*f* rare-earth elements in this energy range, broadly used also in X-ray absorption spectroscopy. Furthermore, oxygen and 3*d* transition metals are the key ingredients of a huge number of materials with intriguing electronic and magnetic properties. Probably the most essential examples for the solid state physics community are cuprate superconductors, layered Cu–O compounds showing superconductivity well above the liquid-nitrogen boiling point at 77 K. That explains why RIXS has been developed mostly in the soft X-ray range, with hundreds of highly cited publications in recent years (Braicovich *et al.*, 2010*a* ▸; Le Tacon *et al.*, 2011 ▸; Moretti Sala *et al.*, 2011 ▸; Bisogni *et al.*, 2012 ▸; Ghiringhelli *et al.*, 2012 ▸; Schlappa *et al.*, 2012 ▸; Dean *et al.*, 2013 ▸; Johnston *et al.*, 2016 ▸; Chaix *et al.*, 2017 ▸; Hepting *et al.*, 2018 ▸; Arpaia *et al.*, 2019 ▸; Revelli *et al.*, 2019 ▸; Rossi *et al.*, 2019 ▸). Moreover, RIXS can be used to study molecules, either in gas, liquid or solid state (Kotani & Shin, 2001 ▸; Hennies *et al.*, 2010 ▸; Yin *et al.*, 2015 ▸; Cheng *et al.*, 2022 ▸). In particular, understanding the oxygen and nitrogen electronic structures is essential for a huge number of substances, whereas organometallic compounds are of particular interest when they contain a 3*d* transition metal. Time-resolved RIXS experiments have been performed at the LCLS, USA, with some remarkable results that established the feasibility and the interest of the technique (Wernet *et al.*, 2015 ▸; Dean *et al.*, 2016 ▸; Mitrano *et al.*, 2019 ▸; Mitrano & Wang, 2020 ▸; Parchenko *et al.*, 2020 ▸; Paris *et al.*, 2021 ▸; Monney *et al.*, 2023 ▸).

### 2.2. Soft X-ray RIXS instrumentation

Due to the low count rate, the first RIXS instruments were based on compact spectrometers, with wide angular acceptance, spherical gratings with constant line spacing mounted in Rowland geometry and microchannel-plate detectors at very grazing incidence (Nordgren & Nyholm, 1986 ▸; Nordgren *et al.*, 1989 ▸; Callcott *et al.*, 1986 ▸). The use of spherical gratings with variable line spacing (VLS) allowed detectors to be mounted at less grazing incidence (Osborn & Callcott, 1995 ▸; Dallera *et al.*, 1996 ▸) and the detector motion range to be reduced, thus allowing longer spectrometers with intrinsic higher resolving power to be designed. With the advent of CCD detectors at affordable prices, good quantum efficiency and sufficiently small pixel sizes, soft X-ray RIXS eventually reached resolving powers better than 2000 in the early-2000s (Dinardo *et al.*, 2007 ▸), and entered the high-resolution age (*E*/d*E* ≥ 10000) in 2007, thanks to the first combined design of the ADRESS beamline and the 5 m-long SAXES spectrometer at the PSI/SLS (Ghiringhelli *et al.*, 2006 ▸; Strocov *et al.*, 2010 ▸). A few years later, larger (8–12 m-long) and more ambitious instruments were built at the TPS (Lai *et al.*, 2014 ▸), ESRF (Brookes *et al.*, 2018 ▸), DLS (Zhou *et al.*, 2022 ▸), NSLS II (Dvorak *et al.*, 2016 ▸) and, for the VUVX range, FERMI@ELETTRA (Dell’Angela *et al.*, 2016 ▸) and FLASH (Dziarzhytski *et al.*, 2020 ▸), or under construction at NanoTerasu (Miyawaki *et al.*, 2022 ▸) and Sirius (Rodrigues *et al.*, 2019 ▸). In most of the cases the spectrometer optical layout is based on VLS spherical gratings, coupled to parabolic collecting mirrors to increase the angular acceptance in the non-dispersive direction.

**Figure 1:**
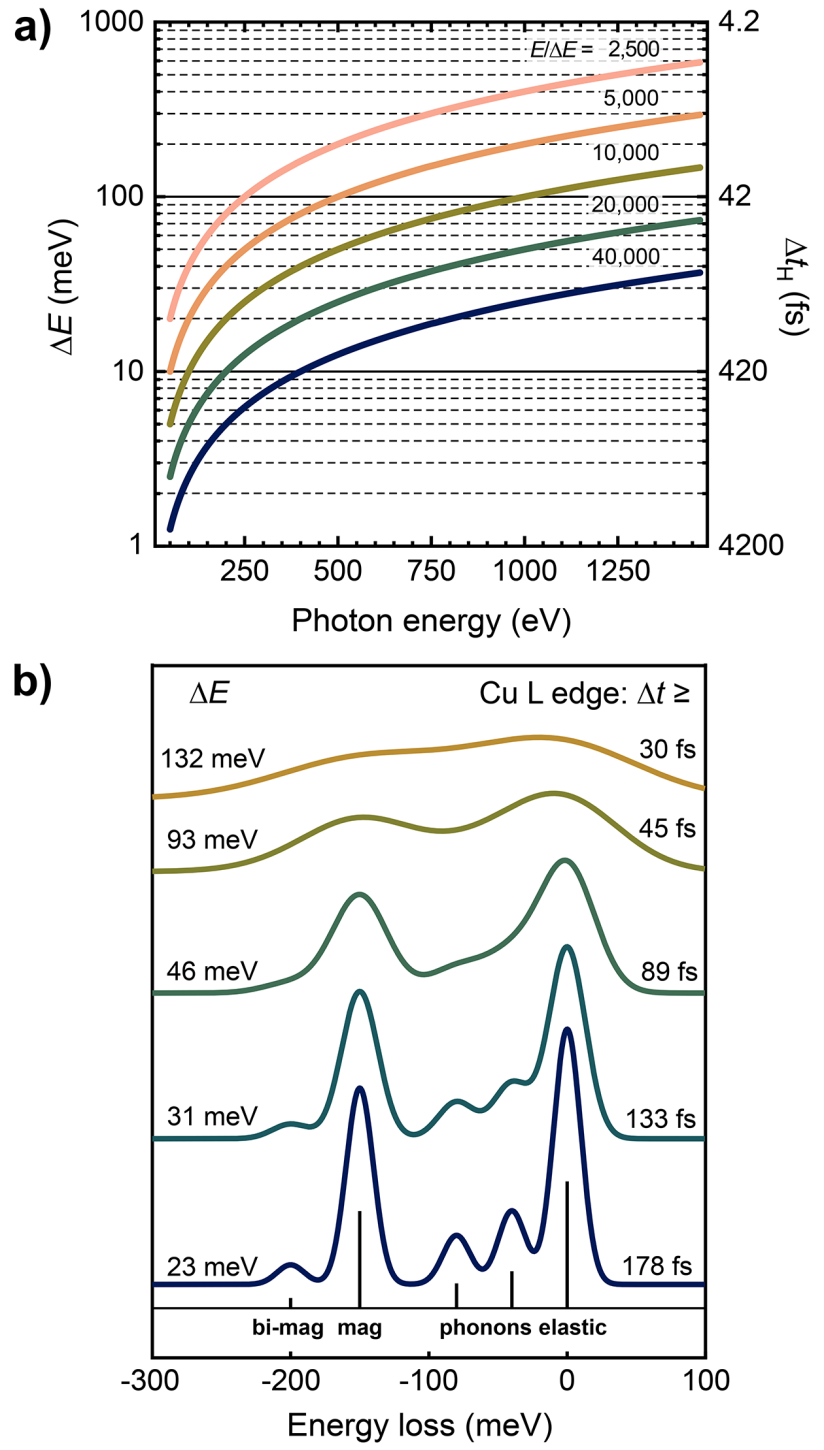
(*a*) The limits set by the Heisenberg uncertainty for time and energy during a soft X-ray spectroscopy experiment. Lower limits for energy and time resolution at given energy resolving power. We notice that a resolution of 30 meV (the present resolution limit for RIXS at synchrotrons at 1000 eV photon energy) sets the time resolution limit to about 138 fs, which is within the scope of SCS (Gerasimova *et al.*, 2022 ▸) and other European XFEL instruments. (*b*) The influence of instrumental energy resolution on the appearance of a hypothetical RIXS spectrum of a correlated copper oxide material (Ament *et al.*, 2011 ▸).

The hRIXS spectrometer was designed to be the first high-resolution spectrometer at a free-electron laser. The goal was to guarantee a maximum flexibility to fulfil the various expectations of the different users. Therefore, it had to allow a very high-energy resolution, matched to that of the beamline, possibly getting close to the Heisenberg limit allowed by the time structure of the FEL. Alternatively, a different configuration based on single-pulse detection and using ultrafast detectors has to be present to push the time resolution at the expense of relaxed energy resolution and larger spot size at the samples. Here, it is interesting to take a look at the theoretical limits of time and energy resolution given by the uncertainty relations, and express them in relation to the resolving power of the instrumentation. A summary of this time–energy landscape and influence on RIXS spectra is presented in Fig. 1 ▸. Moreover, hRIXS should enable the study of multi-photon RIXS-excitations by using a high photon-flux density of the focal spot on the sample. Finally, the facility must allow the continuous change of the scattering angle, and has to be easy to set up and operable with different experimental stations and sample environments.

### 2.3. SCS beamline characteristics

The SCS instrument is one of three soft X-ray beamlines at the European XFEL, located at SASE3 (Tschentscher *et al.*, 2017 ▸). The photon energy of the SCS instrument covers the soft and tender X-ray regime, from 280 eV to 3000 eV. The European XFEL, operating in the SASE regime, produces intense femtosecond pulses up to MHz repetition rate with very high degree of transverse coherence (close to 100%) but limited longitudinal coherence (Geloni *et al.*, 2010 ▸). Within the typical pulse duration of a few femtoseconds to tens of femtoseconds, the coherence time is mostly in the sub-femtosecond range, resulting in tens or hundreds of longitudinal modes. The SASE3 grating monochromator has been designed to substantially reduce the intrinsic bandwidth (0.3–1% in the soft X-ray range) and to improve the longitudinal coherence. Achieving close to transform-limited pulses after the monochromator is a major challenge, limited by the quality of the optical elements, in particular of the grating. Both the figure error and accuracy of VLS spacing increase the time × bandwidth product with respect to what is expected for ideal optics. In the design, transmission > 4σ of the Gaussian beam profile is foreseen, requiring particularly long optics. This will allow the diffraction limit to be approached whenever long gratings of supreme quality become available (Gerasimova *et al.*, 2022 ▸). At the moment two gratings of much shorter length have been implemented. The low-resolution grating is optimized for time-resolved experiments (a few to a few tens of femtosecond RMS) and moderate resolving power of 2000–5000. The high-resolution grating reaches a resolving power of 10000 at the cost of larger pulse stretching in time (by about a factor of three). While the time × bandwidth product of these gratings is estimated to be close to ideal below 500 eV, it is increased at higher photon energies, due to the reduced transmission of the beam profile (Gerasimova *et al.*, 2022 ▸). The total transmission of the beamline is in the range 10^−4^–10^−3^ and results in a pulse energy between 0.1 µJ and 10 µJ at the sample. Details of the SASE3 monochromator are given by Gerasimova *et al.* (2022 ▸). The focus size of the FEL beam at the SCS instrument is variable, controlled by Kirkpatrick–Baez optics. The bendable mirrors allow the vertical and horizontal focus to be changed independently from 1 mm down to ∼1 µm (Mercurio *et al.*, 2022 ▸). For RIXS studies a horizontally elongated beam is particularly convenient, with a small vertical size (typically ∼10 µm) and 100–500 µm horizontally, matched to the pump laser beam size at the sample.

## 3. Design of the spectrometer and its environment

### 3.1. Constraints for the design

Several constraints had to be taken into account for the design and construction of the hRIXS spectrometer. The experimental hall of the European XFEL (where all instruments are located) is placed underground. This puts severe limitations on the available space, meaning that the total length of the hRIXS spectrometer could not exceed 5 m. The range of possible positions of the refocusing focal point combined with the size of the experimental hutch impose the range of the accessible scattering angle 2Θ to be 65–145° (an overview can be found in the the supporting information). Moreover, the spectrometer cannot be permanently installed, because the SCS instrument hosts a range of interchangeable setups dedicated to different experiments. Therefore, the hRIXS spectrometer has been designed to be retractable while at the same time minimizing the time for putting it on- and off-line, and, even more importantly, to properly align it and start measuring, so as to make the best use of the beam time. In addition, the hRIXS spectrometer has to be coupled to different interaction chambers and sample environments, *e.g.* a goniometer mounting for solid single crystals or a liquid-jet setup for molecular systems.

The European XFEL operates in burst mode at 10 Hz. Every 0.1 s a burst of pulses is emitted during a time window of 600 µs. Up to 400 µs of that time can be dedicated to SASE3, that would result in a train of pulses separated by a minimum interval of 885 ns. Therefore, one long pulse train can deliver up to 452 FEL pulses at the rate of 1.13 MHz to SASE3 and up to 4520 pulses s^−1^. Due to the SASE process, the spectral distribution and the intensity vary widely from pulse to pulse. Advanced diagnostics at the European XFEL allow measurements of single-shot and average pulse intensities for data normalization (Maltezopoulos *et al.*, 2019*a* ▸).

This effective repetition rate is extremely demanding for detectors, particularly when combined with high spatial resolution, large area, and large number of pixels as for RIXS spectrometers. There are three options for operation, which are given by the readout time of the detector: (i) pulse-resolved detection; (ii) train-resolved detection; and (iii) integrating detection. Pulse-resolved detection requires high readout time (1 µs range or better). In addition, the large amount of data from a two-dimensional detector requires data-storage capabilities, as the data cannot be sent out at that rate. Pulse-resolved detection would be the preferred choice, giving the possibility to normalize the data shot-by-shot, to take reference data in parallel, and to allow the highest temporal resolution to be achieved. However, at present there are only few detectors that operate at that rate and they have moderate spatial resolution. Examples are based on a microchannel-plate photon converter and delay-line current pulse detection with position encoding, as well as mega-pixel-size detectors built of Si-sensors (Porro *et al.*, 2021 ▸). Train-resolved detection requires moderate reading and transfer speed (better than 100 ms). The temporal resolution is limited by the jitter within each train (that is already smaller than jitter between single trains and considerably smaller than FEL pulse stretching due to the monochromator). Examples of detectors that can currently work in that mode are based on electron-amplified CCD and CMOS technology. Any detector that requires acquisition times of 1 s or longer has to be operated in integration mode. The ‘standard’ high-resolution detectors, CCDs, have to be operated in that mode.

### 3.2. Optical layout

We have worked to find the best trade-off in terms of throughput, resolution and operative flexibility given the diverse and often contrasting needs of the different operation modes foreseen for the hRIXS instrument. For the study of low-energy excitations in solid samples the highest resolving power is the priority, whereas in other cases the temporal resolution represents the limiting factor and energy resolution can be compromised. The former has implications on the choice of detector (fast detectors capable of resolving single X-ray pulses have poor spatial resolution with respect to the CCD/CMOS detectors that necessarily integrate over thousands of X-ray bunches). Moreover, the beam spot size on the sample has to be tunable to adopt the X-ray beam size to the sample size (*e.g.* for different liquid jets) and the focal size of the pump laser that can vary strongly with wavelength and required pulse energies. This allows a trade-off to be found for possible high signal level, while staying below the damage threshold of particularly solid samples by X-ray and pump laser beams. The overall X-ray intensity can be decreased by tuning a gas attenuator located in the SASE3 tunnel.

We have initially explored the performances of a single VLS spherical-grating spectrometer over an extremely wide range of parameters. This elementary optical layout has several advantages for an instrument of about 5 m in total length. The total range of positioning of the detector is easily manageable with standard mechanical actuators and the whole instrument can be held on a single girder, while keeping the internal alignment. The mechanical stability is provided by the high-quality floor of the experimental hutch. A single or double parabolic mirror usually added in longer spectrometers [ERIXS at ID32/ESRF (Brookes *et al.*, 2018 ▸), I20 at DLS (Zhou *et al.*, 2022 ▸), SIX at NSLS II (Dinardo *et al.*, 2007 ▸)] would bring a relatively small gain in luminosity, at the cost of relevant mechanical additional complications in the small available space and would not work properly with a horizontally wide beam to be adopted in most of the experiments (the parabolic mirrors cannot work properly with a millimetre-sized source). Therefore, having fixed the maximum length (sample-to-detector distance) given by the physical space available in the SCS experimental hutch, over a wide range we have explored the four key parameters of the spectrometer:

(i) The vertical spot size on the sample surface, *S*_1_.

(ii) The actual spatial resolution for the 2D position-sensitive detector, parallel to the detector surface, *S*_2_.

(iii) The central grating ruling density, *a*_0_.

(iv) The entrance arm length, *r*_1_.

**Figure 2:**
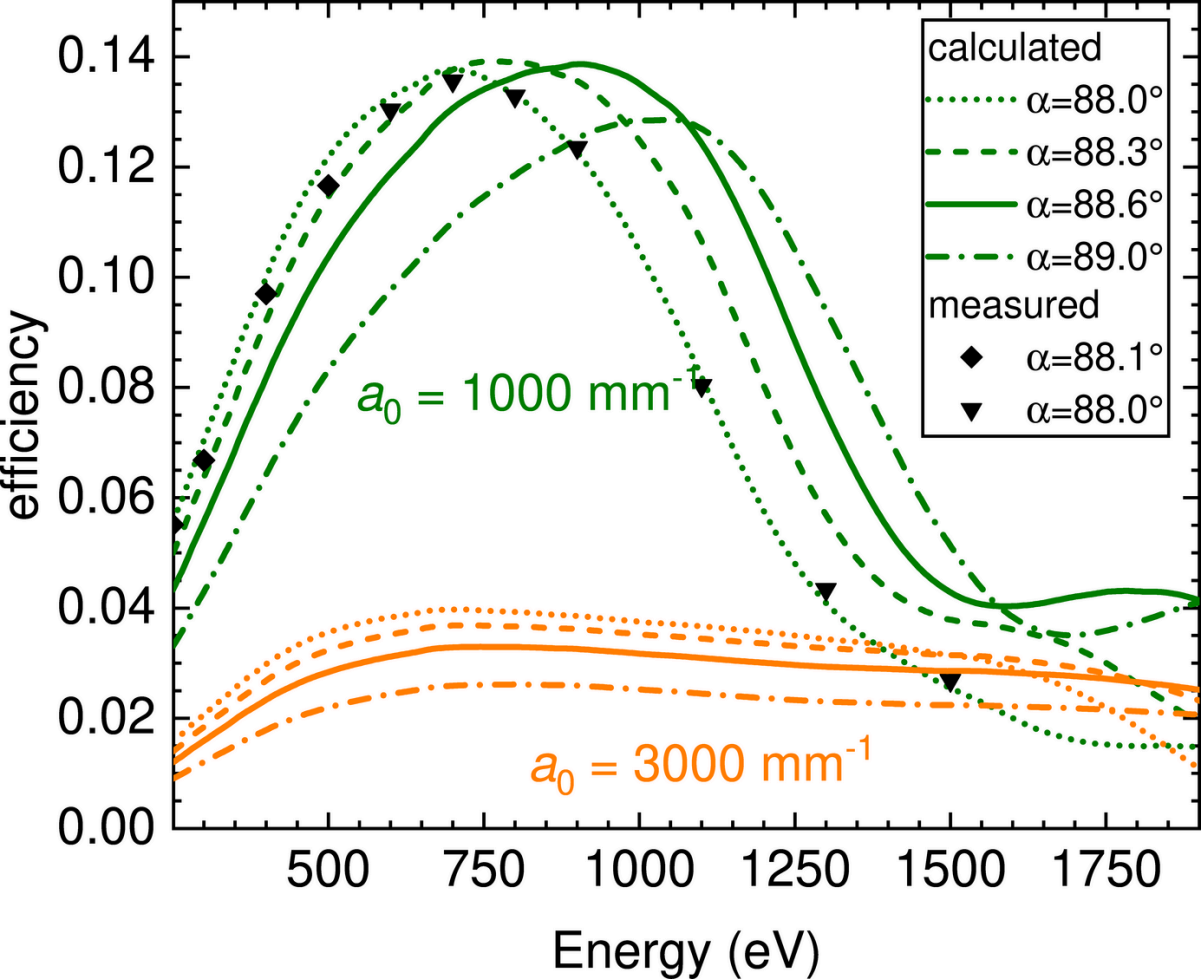
hRIXS spectrometer grating efficiency. Lines represent the computed values at four incidence angles with Au coating, aspect ratios *c*/*d* = 0.60 and 0.65, and groove depths *h* = 5 nm and 9 nm for the high-resolution grating (HRG) and high-transmission grating (HTG), respectively (Schäfers & Krumrey, 1996 ▸). The measured values for the HTG are shown by symbols (measurement performed at PM1 at BESSY II).

The other parameters (grating radius of curvature, *R*; VLS polynomial coefficients of the ruling density, *a*_1_, *a*_2_, *a*_3_; angle of incidence on the grating, α; focusing conditions, with cancellation of coma aberration, *r*_2_ and β) are calculated following the procedure used by Ghiringhelli *et al.* (2006 ▸) with the corrections added by Strocov (2010 ▸). The (grazing) angle of incidence on the detector, γ, is fixed; for the calculations a reference value is used, typically around 20°, for which the effective spatial resolution of the detector is improved by a factor 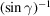 ≃ 4.4 and the quantum efficiency of Si-based detectors (CCD or CMOS) is still close to 1. The analytical calculations were then cross-checked and refined with the *Shadow* ray-tracing code (Sanchez del Rio *et al.*, 2011 ▸) and with the code of Strocov for spherical VLS-based spectrometer design (Strocov *et al.*, 2011 ▸). The boundary conditions were set by the maximum total length (*L* = 5000 mm) and by the minimum *r*_1_ = 900 mm. Two gratings (*a*_0_ = 3000 mm^−1^ and *a*_0_ = 1000 mm^−1^) are sufficient to cover the whole working energy range 250–1600 eV with good flexibility. After a preliminary comparison of the performances of spectrometers optimized on five different combinations of source size and detector effective spatial resolution, we have selected *S*_1_ = 5 µm and *S*_2_ = 10 µm for the final optimization. The choice of the two groove densities was dictated by the need of maximizing dispersion, while keeping a decent efficiency (*a*_0_ = 3000 mm^−1^, first-order diffraction efficiency better than η > 3%) in one case, and to maximize efficiency with a reasonable resolving power (*a*_0_ = 1000 mm^−1^, η > 12%) in the other case (see Fig. 2 ▸).

**Figure 3:**
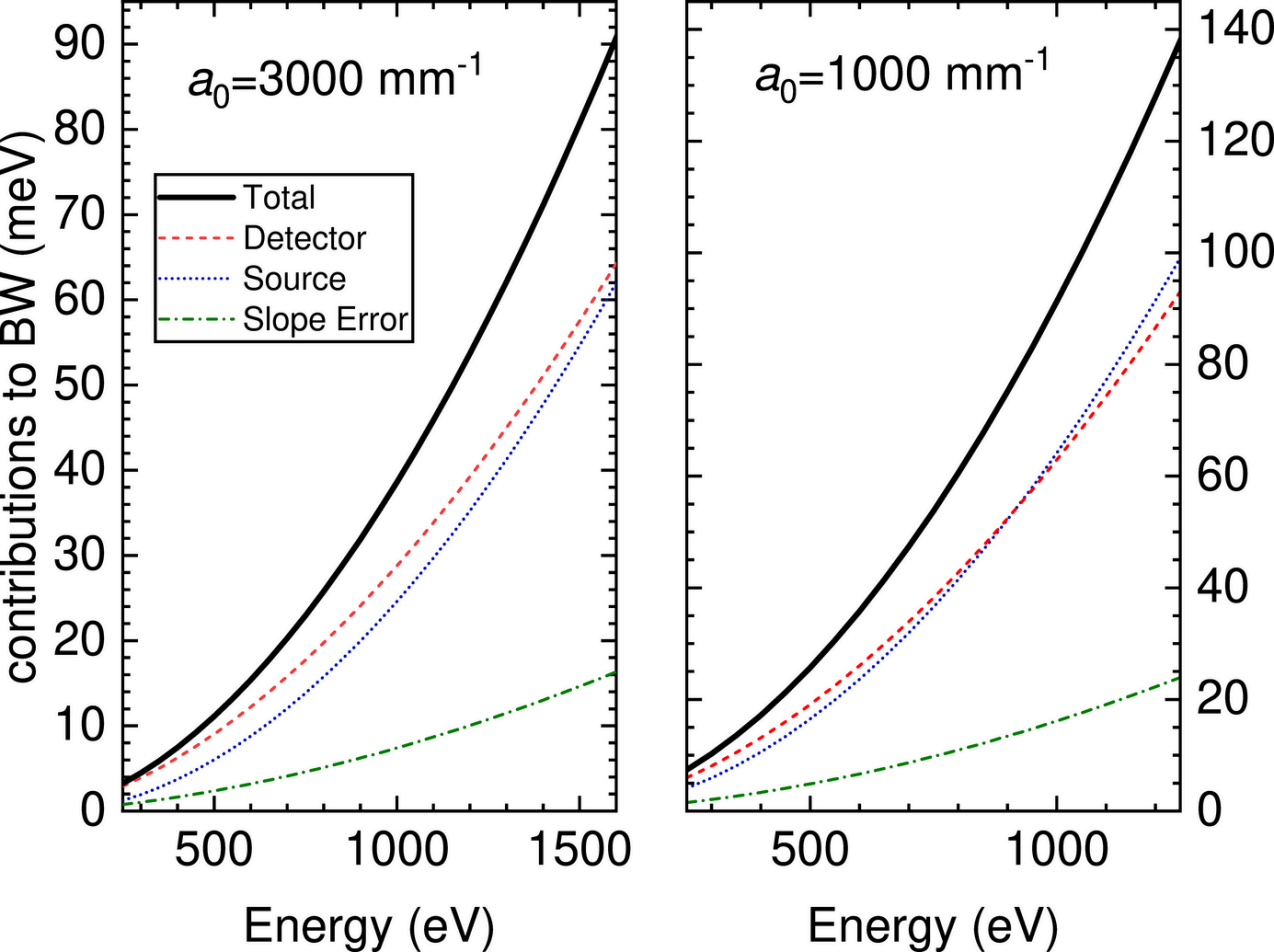
Contributions to the spectrometer energy bandwidth for the two gratings. The total resolution is given by the quadratic combination of the partial values. Calculations made for the nominal values of source size and detector spatial resolution used for the optimization of the VLS parameters (*S*_1_ = 5 µm, *S*_2_ = 10 µm) and slope error *s*′ = 0.1 µrad r.m.s.

The VLS parameters *R*, *a*_1_ and *a*_2_ were optimized using the criterion that, at a reference energy *E*_0_, the contributions to the total spectrometer bandwidth coming from *S*_1_ and *S*_2_ are equal (see Fig. 3 ▸). This balance is respected over the whole energy range, having fixed the reference total length *L*_0_ = *r*_1_ + *r*_2_ and the grazing-incidence angle on the detector γ = 20°. This optimization, also used for the ESRF instrument (Brookes *et al.*, 2018 ▸), is made analytically as described in detail in the supporting information, and eventually checked by ray-tracing simulations. For the optimization we chose to have *S*_2_ = 2*S*_1_. In the VLS parameter optimization process it is important to check that the full energy range is covered while fulfilling the coma aberration minimization criterion. The eventual parameters are summarized in Table 1 ▸.

As shown in Fig. 3 ▸, this design has many excellent features. The resolving power is higher than 30000 (20000) for photon energies below 1000 eV (500 eV) using the high-resolution grating, HRG, with *a*_0_ = 3000 mm^−1^ (high-transmission grating, HTG, with *a*_0_ = 3000 mm^−1^). The optimization of performances requires a good optical matching between the beamline and the spectrometer, and is obtained with a minimum of optical components and an entrance-arm length that varies little with energy. The resolving power obviously decreases with larger spot size and worse detector resolution. A slope error *s*′ = 0.1 µrad r.m.s. for the grating has been considered all along the calculations. This value is extremely good, at the limit of the present technology for spherical gratings. However, as will become evident below, a less stringent slope error (up to 0.25 µrad r.m.s.) would not alter significantly the resolution of the proposed optical layout.

**Figure 4:**
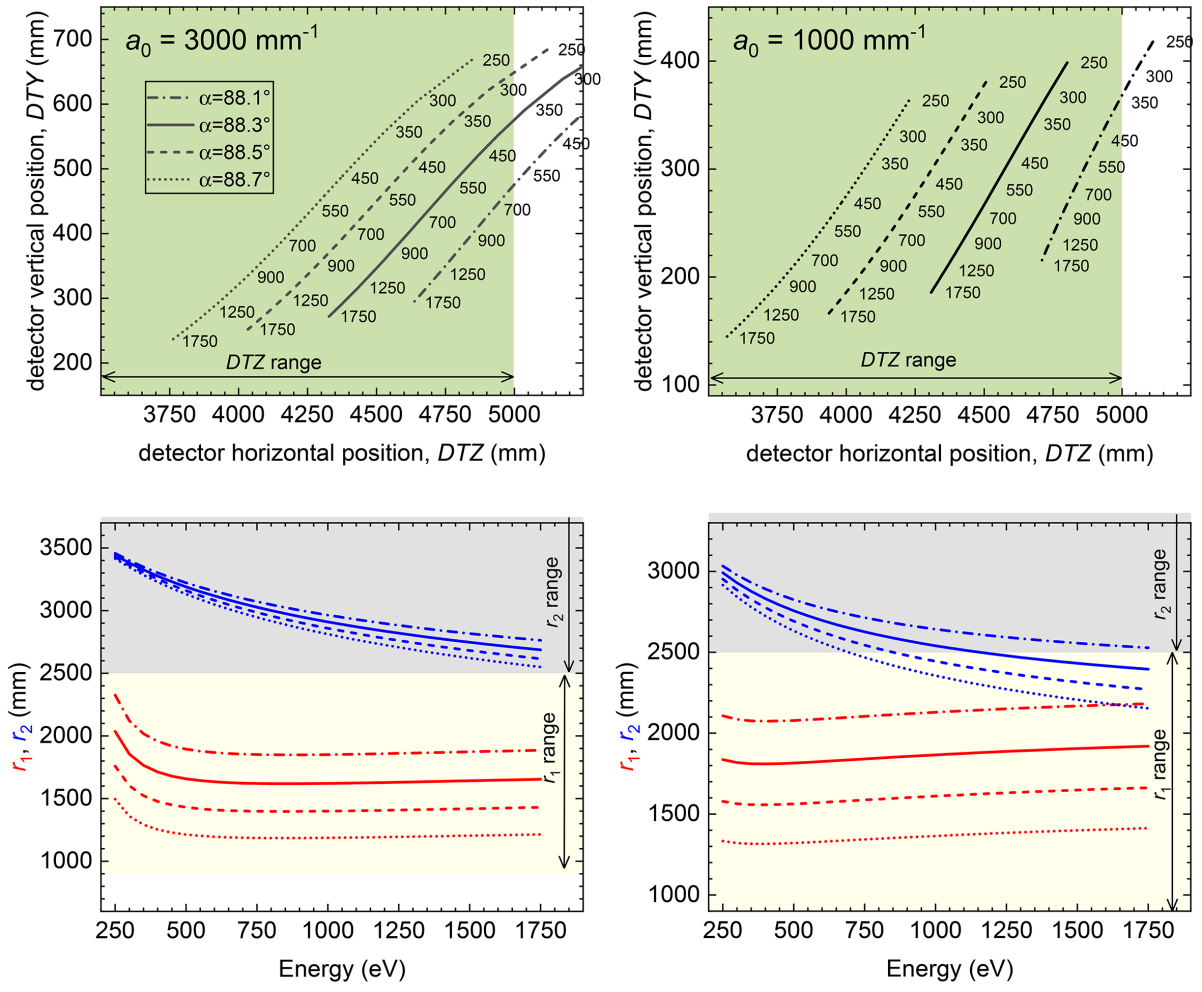
Representation of the hRIXS spectrometer working points for the HRG (left) and the HTG (right). The graphs at the top show the accessible photon energies for a fixed incident angle α and the corresponding detector position. The bottom graphs show entrance and exit arm, *r*_1_ and *r*_2_, for a given photon energy and incidence angle α.

**Figure 5:**
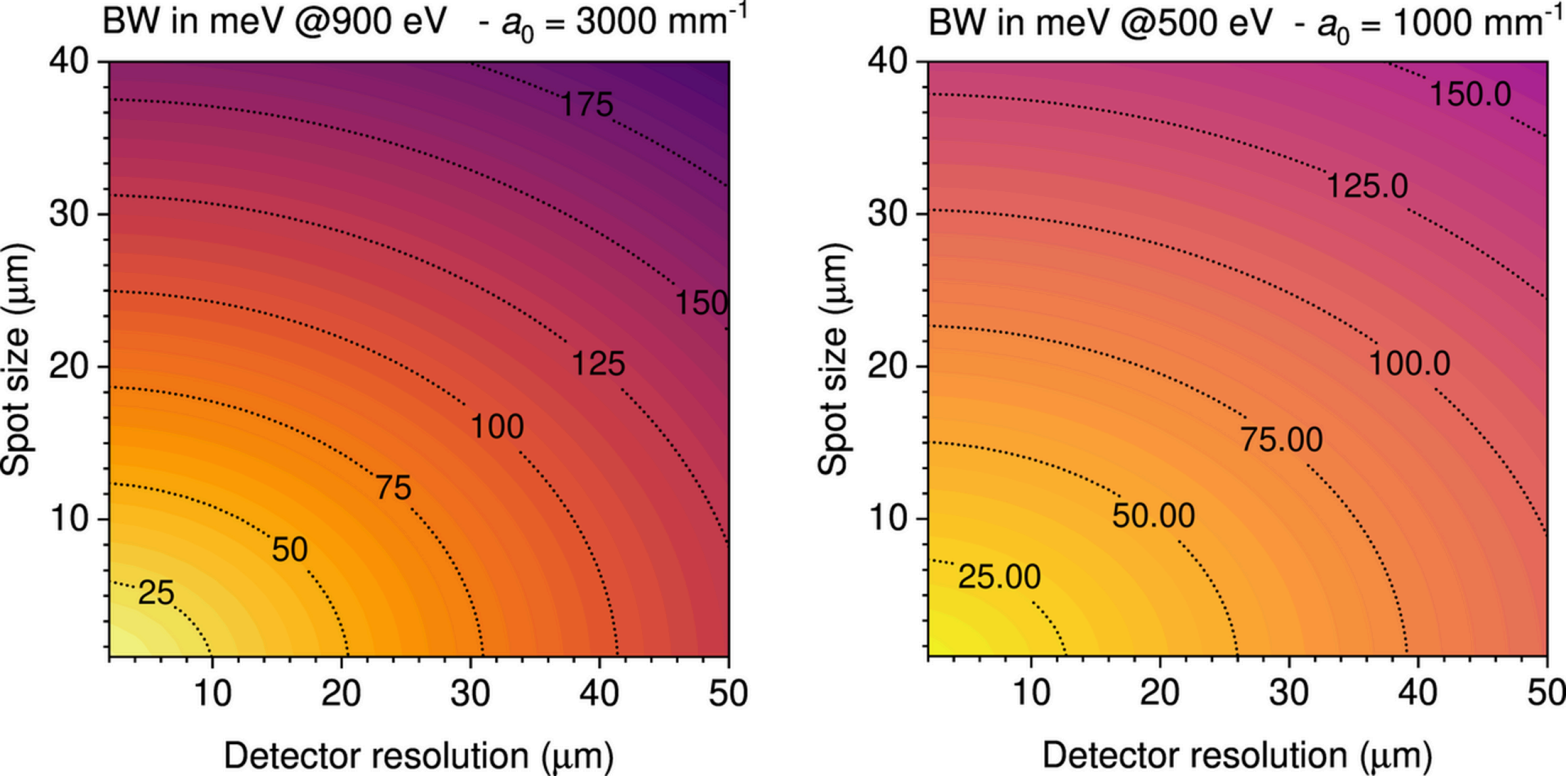
Spectrometer energy bandwidth for the two gratings as a function of spot size *S*_1_ and of detector spatial resolution *S*_2_. The total resolution is given by the quadratic combination of the partial values. Calculations made for the nominal value of the slope error *s*′ = 0.1 µrad r.m.s.

For all gratings a laminar trapezoidal groove profile was made on single-crystal silicon substrates with final reflective Au coating. The high-transmission grating was produced by Precision Gratings (HZB) (Siewert *et al.*, 2018 ▸) on a substrate from Pilz (measured sagittal slope error *s*′ = 0.11 µrad r.m.s.). The high-resolution grating was produced by HORIBA Jobin Yvon on a substrate from Zeiss (*s*′ = 0.18 µrad r.m.s.). The deviation of the VLS parameters from specifications was smaller than 2% (up to the second order). Both gratings can cover the 250–1750 eV photon energy range with the appropriate choice of the (α, *r*_1_) parameter combination that cancels the coma aberration, as shown in Fig. 4 ▸. The HRG, optimized for the *L*_2,3_ edges of 3*d* transition metals, can be used with 88.1° ≤ α ≤ 88.7° for every energy above 600 eV. The HTG, designed for the lower energy range, can be used with 88.2° ≤ α ≤ 88.7° already from 250 eV. The best choice of (α, *r*_1_) will be determined case by case, optimizing the actual efficiency and resolution. Those depend also on other parameters, such as the beamline monochromator, the beam size on the sample, and the detector performances. An overview of the influence of *S*_1_ and *S*_2_ on the final resolution is shown in Fig. 5 ▸.

A third grating for the tender X-ray energy range (from 1500 eV to 3000 eV) is foreseen with a dedicated grating holder. Calculations indicate that a resolving power better than 15000 and efficiency larger than 1.2% for *a*_0_ = 3000 mm^−1^ and α ≃ 89.0° are possible over the whole range.

### 3.3. Mechanical design

**Figure 6:**
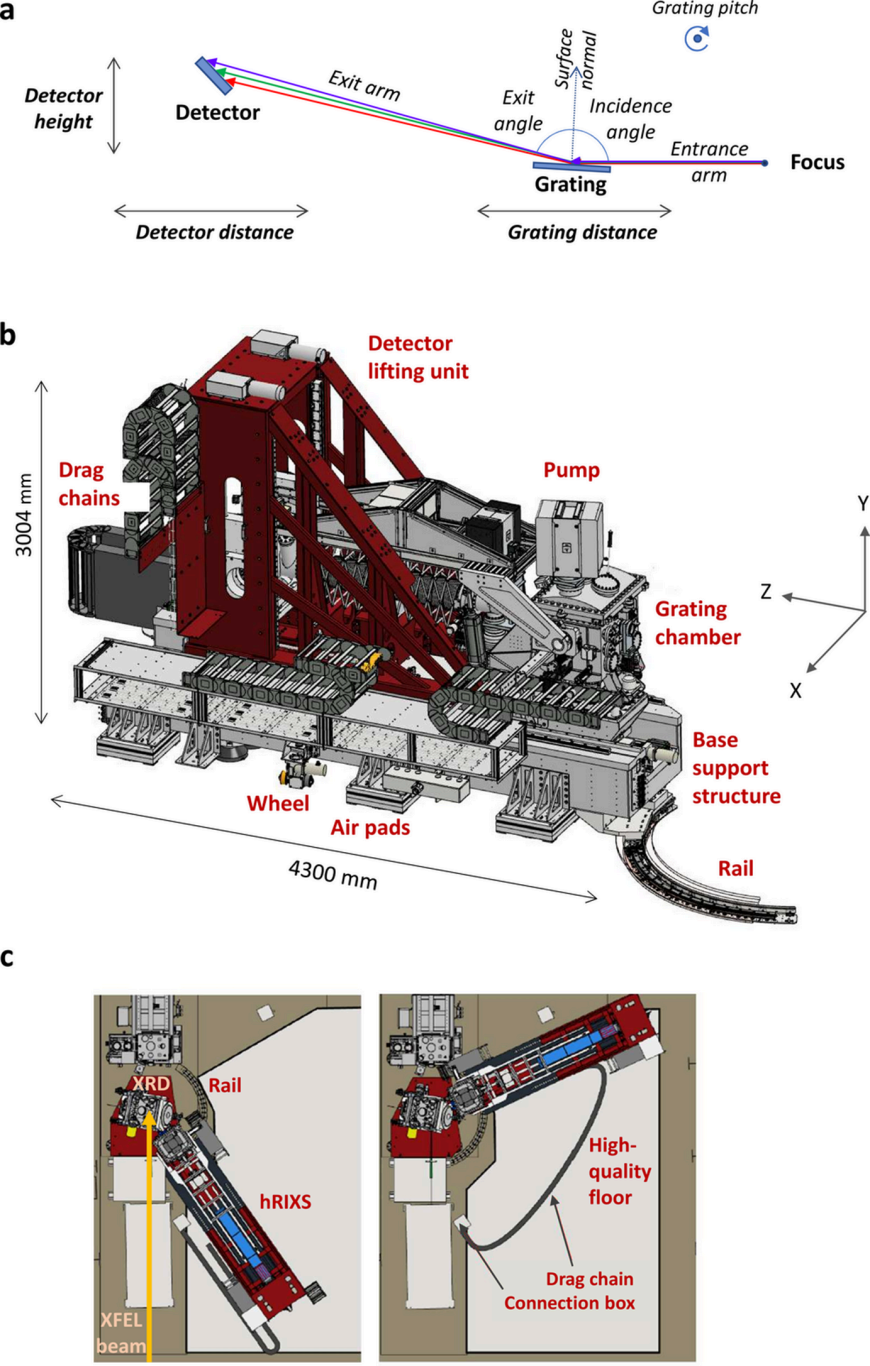
hRIXS spectrometer overview: diffraction scheme and degrees of motion for operation (*a*), model of the spectrometer (*b*), top view of the spectrometer when placed at the interaction point at the two extreme scattering angles: back-scattering at 2Θ = 145° (left) and forward-scattering at *t*Θ = 65° (right) (*c*).

There are two optical elements inside the hRIXS spectrometer – the grating and the detector. This is the minimum number of elements required for adjustment of a spherical VLS grating spectrometer. Since the spectrometer had to be easily adjustable, a collecting mirror was not foreseen. In order to align the spectrometer for one photon energy, four degrees of freedom are required: the incidence angle on the grating α, the entrance arm length *r*_1_, the exit angle β, and the exit-arm length *r*_2_. These are realized by the following four motions: grating pitch angle (changing the incidence angle α), horizontal translation of the grating chamber (changing the distance from the grating to the focus *r*_1_), horizontal translation of the detector chamber, and the vertical translation of the detector chamber (both changing the distance between the grating and the detector *r*_2_ and the exit angle β), see Fig. 6 ▸(*a*). The required movement range for the translation of the two chambers is large (around 1.5 m), to allow operation of the spectrometer over a large photon energy range with one grating. At the same time the stability requirements are extremely high (in the micrometre range). The working points of the spectrometer are summarized in Fig. 4 ▸. Table 2 ▸ displays the motion range (see also the supporting information for stability measurement data).

**Figure 7:**
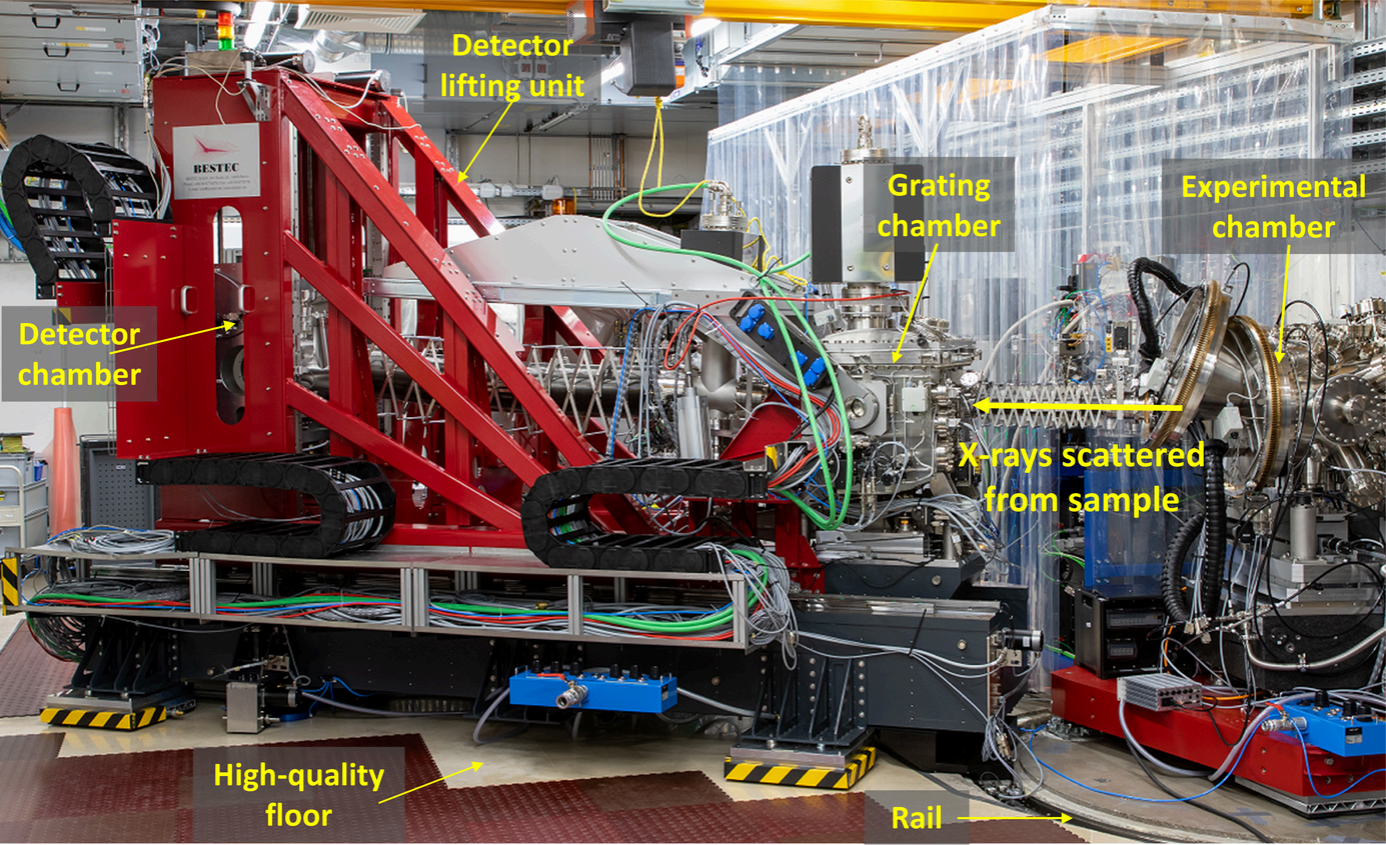
Photograph of the hRIXS spectrometer installed in the working point inside the SCS hutch (the high-quality floor is partially covered for protection).

The model of the spectrometer is shown in Fig. 6 ▸(*b*). The spectrometer is placed on air pads on top of a high-quality floor. The bottom part of the support consists of a large mineral casting base, to ensure very good thermal and mechanical stability (for more details see the supporting information). The grating chamber has an additional mineral casting support, which also holds the inner mechanics. The detector chamber is held by a steel lifting. When the spectrometer is placed at the interaction point, rails inside the experimental floor guide the rotation of the spectrometer in order to scan the scattering angle. Fig. 6 ▸(*c*) shows spectrometer positions for the maximum and minimum scattering angles. When not in use, the hRIXS spectrometer can be detached and placed in a parking position, away from the interaction point. The weight of the spectrometer is approximately 10 t and the dimensions are roughly 4.2 m × 2.1 m × 3.1 m (L × W × H). Fig. 7 ▸ shows a photograph of the hRIXS spectrometer when connected to the interaction point.

Due to the small vertical X-ray spot size at the SCS instrument, there is no need for an entrance slit for the hRIXS spectrometer. A mask unit, placed in front of the grating, gives the possibility to narrow down grating illumination. The inner mechanics of the grating chamber provides space for three gratings. Exchange of gratings is realized by a horizontal transverse motion. There are additional degrees of motion for grating alignment. Behind the grating tank an aperture unit with four independent aperture blades can be used to shield stray light falling on the detector. The detector chamber is optimized for large detectors. The detector mounting flange has the size of 300 diameter nominal (DN300) and is tilted at γ = 25° to the exit arm. In order to keep the incidence angle fixed at the detector and also to prevent the large bellows connecting the grating chamber with the detector chamber from breaking, the detector chamber needs to be rotated when changing the detector position.

### 3.4. Controls

Using the Karabo control system (Hauf *et al.*, 2019 ▸), the movement of the spectrometer is carried out in the physically relevant coordinates (*i.e.* entrance- and exit-arm distances and angles) instead of individual motor positions. It also assures that the spectrometer does not move outside of its safe working parameters. The movement of the scattering angle involves lifting and rotating the spectrometer, which needs to be followed precisely by the motion of the endstation in order not to break the connecting bellows. All motions are orchestrated by the Karabo control system, while the real-time parts are written using *TwinCat3* by Beckhoff Automation. The same control system is also used to automate standard experimental procedures, like θ–2Θ scans, knife-edge scans or pump–probe delay scans.

## 4. Sample environment

### 4.1. Pump–probe laser

The facility pump–probe (PP) laser system was developed to operate in the same mode as the FEL (burst mode at 10 Hz) (Pergament *et al.*, 2016 ▸). Two fundamental wavelengths – femtosecond-pulse 800 nm and long-pulse 1030 nm – are foreseen. We are currently working with the first, while the second is being commissioned. There are two operation modes: 15 fs and 50 fs (FWHM). Four working points for the repetition rate are used at SCS: 1.13 MHz, which can deliver pulse energies up to 0.2 mJ for the 800 nm pump, as well as 565 kHz, 188 kHz and 113 kHz, which can deliver factors of two, six and ten higher pulse energies, respectively. Depending on future experimental needs, further modes might be implemented. Besides 800 nm, these pump wavelengths are currently available: 400 nm (second harmonic generation), 266 nm (third harmonic generation) and wavelengths in the range 2.5 µm down to 350 nm [that can be generated using an optical parametric amplifier (OPA). Details on the PP laser system can be found in Pergament *et al.* (2016 ▸). The minimum laser spot size at SCS is in the 100 µm range (depending on wavelength and the focal distance). Linearly (variable) and circularly polarized laser pump is available. There is a laser-incoupling unit placed 2 m upstream from the interaction point that is available for nearly collinear incoupling. Incoupling schemes at shorter focal distance are available as well. An advanced synchronization scheme for the FEL accelerator and the facility PP lasers minimizes the X-ray/optical timing jitter to the low tens of femtoseconds (Schulz *et al.*, 2015 ▸; Schulz *et al.*, 2019 ▸).

### 4.2. Chemical sample environment

**Figure 8:**
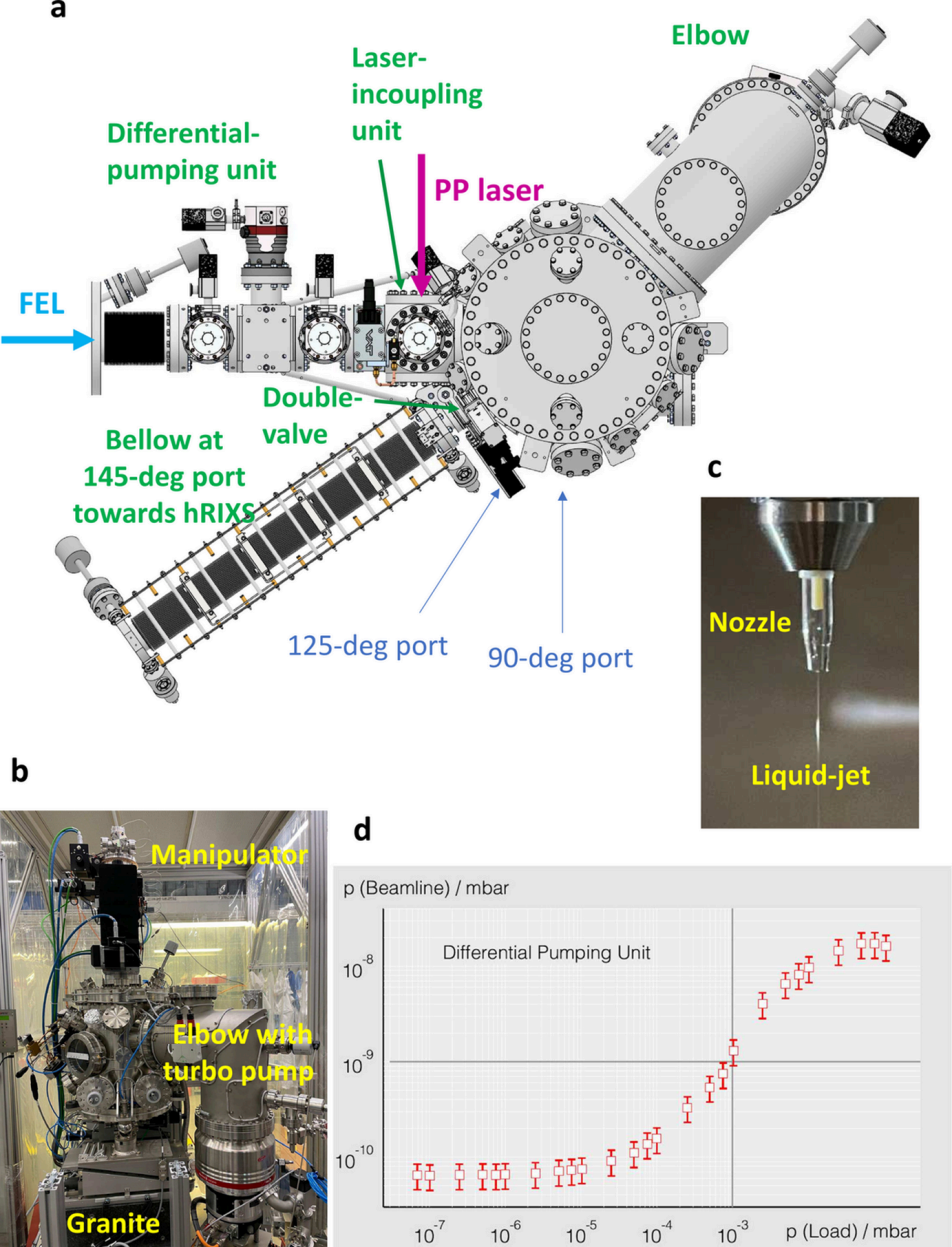
CHEM setup. Model with top view (*a*), photograph of the CHEM chamber (*b*), photograph of the liquid jet (*c*), and performance of the differential pumping unit (*d*).

Time-resolved pump–probe RIXS experiments of chemical systems in their natural environment (the liquid phase) is enabled in the experimental CHEM endstation shown in Fig. 8 ▸. This poses several technical requirements in terms of the sample-delivery system, photon-diagnostic tools and vacuum conditions.

The experimental main chamber is a DN300 tube with CF300 flanges on the top and bottom, CF150 entrance flange towards the beamline, and CF40 connections to the hRIXS spectrometer in 2Θ = 90°, 125° and 145° scattering angle with respect to the incoming FEL beam. For the installation of optics, diodes and other experimentally relevant devices, a board with M6 threaded holes is mounted inside the chamber. A CF250 flange connects the pumping unit to the main chamber with a HiPace 2300 turbomolecular pump. Two cold traps are installed for the collection of the liquid sample and improving the vacuum during operation. The whole endstation sits on an adjustable support structure with five degrees of freedom for an optimal alignment of the FEL beam through the apertures of the differential pumping stage (DPS). The sample manipulator is placed on the top CF150 flange of the CHEM vessel and consists of DDF 63 rotary feedthrough and PMM 12 *XYZ*-manipulator from VAb (the feedthrough is mounted above the manipulator). Table 3 ▸ shows an overview of the available degrees of freedom for the sample motion.

The liquid sample is injected into the interaction point via the liquid-microjet technology consisting of an HPLC pump from Shimadzu and a quartz capillary nozzle with an orifice of the order of around 15 µm up to 50 µm. A switching device allows up to six different samples to be changed. The custom-designed nozzle holder is part of a multipurpose unit, which additionally includes tools for diagnostics at the interaction point (to check the FEL and laser beam size via the knife-edge method, fluorescence screens to optimize the spatial overlap between laser and FEL beams and a diode as well as an Si_3_N_4_ membrane for finding the laser-FEL time overlap). The holder is also suitable for mounting solid samples for studies with the CHEM setup.

The high vapour pressure of liquid samples and the strict vacuum conditions of the beamline require a highly efficient DPS to overcome the several orders of magnitude in vacuum difference, which is connected between the last valve of the SCS beamline and the gate valve before the laser incoupling chamber. With a running liquid jet the usual pressure in the main chamber is of the order of 10^−3^ mbar and the beamline interlock is around 10^−8^ mbar. To accomplish the vacuum difference, the DPS consists of three pumping units, each with an aperture of diameter 2.5 mm, made from B_4_C. The DPS can overcome a pressure difference of more than five orders of magnitude. The vacuum of the hRIXS spectrometer is protected by a membrane that is mounted on the double valve at the CHEM vessel facing the hRXIS connection.

The laser incoupling unit consists of a CF100 chamber with six ports and is connected to the entrance of the main chamber and a gate valve to the DPS. It accommodates a motorized 2-inch mirror mount, which itself can be adjusted by a motorized *XYZ*-manipulator. An aperture with a rectangular shape shields the optics from the spraying of the liquid jet.

### 4.3. Solid sample UHV environment

An ultra-high-vacuum (UHV) environment down to 10^−9^ mbar is provided by the SCS instrument baseline chamber for X-ray resonant diffraction (XRD). This setup is equipped with a diffractometer for time-resolved and non-linear XRD studies. It offers a cryogenic sample environment for solid samples in the temperature range from ambient down to 16 K.

**Figure 9:**
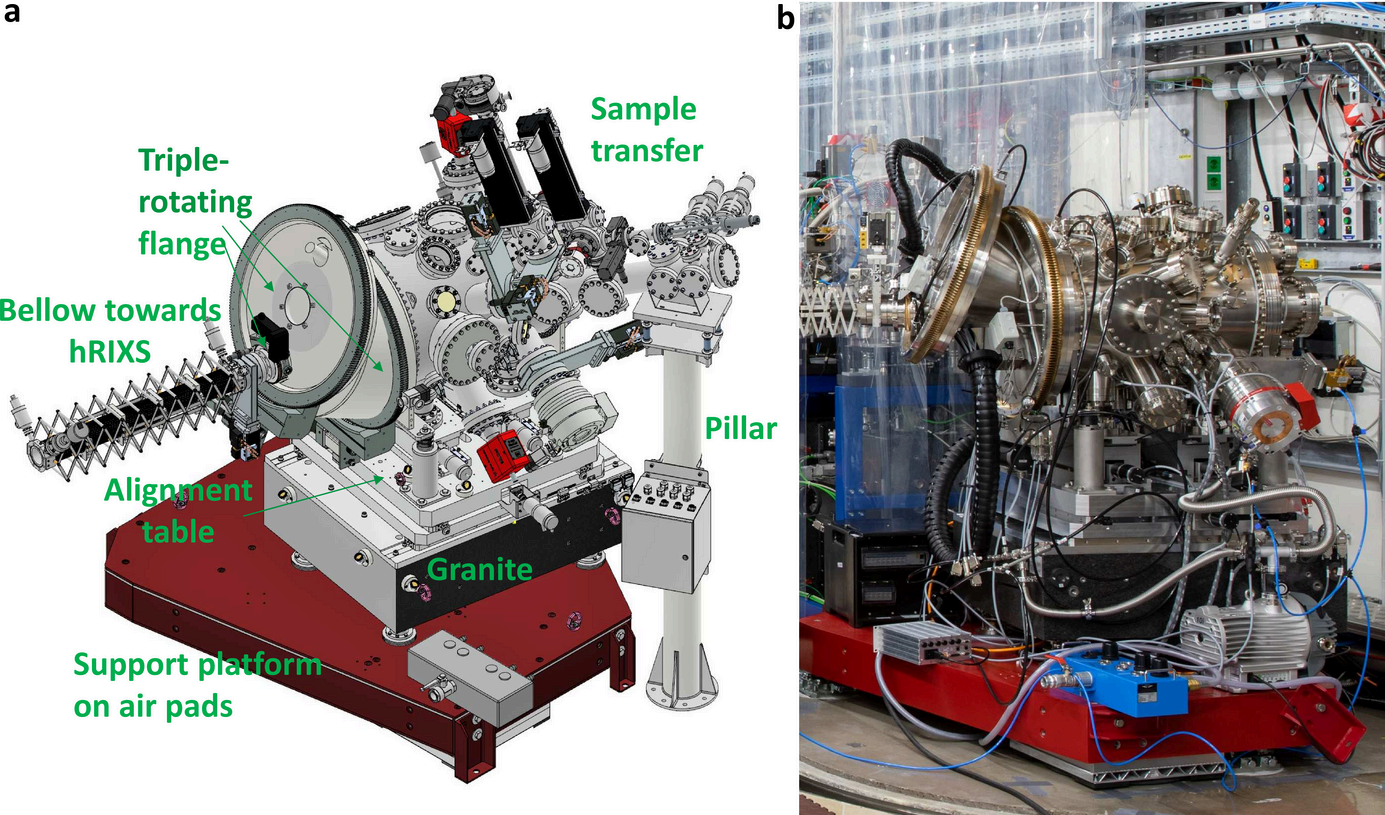
Overview of the XRD setup for a solid sample environment: model (*a*) and photograph (*b*).

Fig. 9 ▸ shows an overview of the setup from the outside. The core piece of the XRD vessel is the triple-rotating flange (TRF), which is the connecting flange to the hRIXS spectrometer. The TRF consists of three flanges, each one connected to a rotary feedthrough. Through a combined motion of the feedthroughs the connecting flange can move in the horizontal plane by about 90°, which allows a continuous change of the scattering angle (see also the supporting information). Besides the hRIXS spectrometer, the flange can be alternatively connected to a 2D X-ray detector.

The XRD chamber is placed on a support platform with air cushions (as is also the CHEM chamber), allowing to displace and place it back at the interaction point with precision below 100 µm. The chamber is supported by a granite block which ensures good thermal and mechanical stability. A horizontal table above allows the chamber to be aligned along and perpendicularly to the X-ray FEL beam. Vertical alignment is carried out by the inner mechanics placed on wedge shoes. The XRD setup is equipped with a sample transfer system.

**Figure 10:**
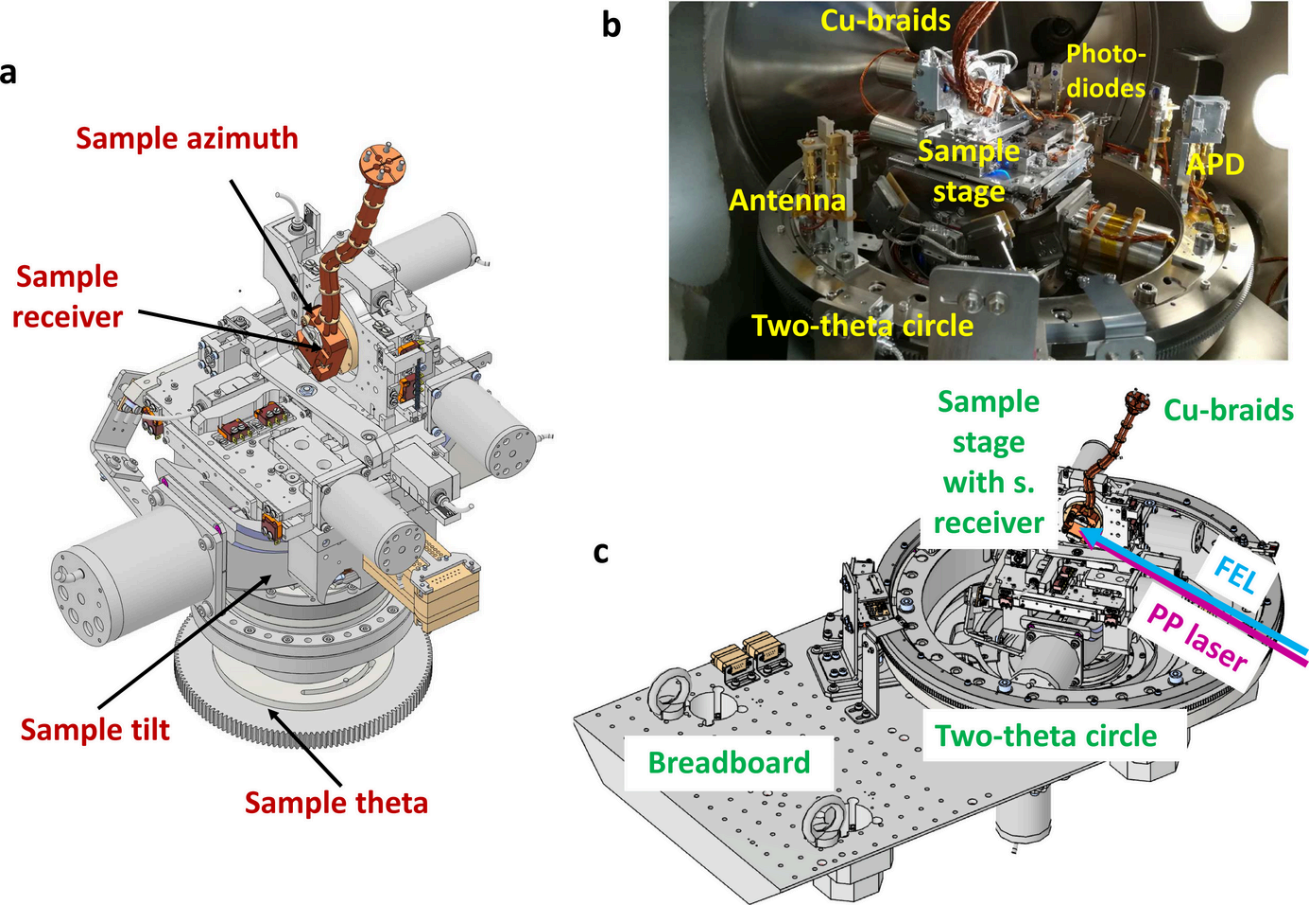
Inner mechanics of the XRD chamber: model revealing degrees of motion for the sample (*a*), photograph showing the diffractometer and the sample stage (*b*), and model with overview of the inner mechanics (*c*).

The inner mechanics consist of a diffractometer, a sample stage and a breadboard (see Fig. 10 ▸). These parts form one entity that is supported directly by the wedge shoes and decoupled from the XRD vessel, making the entire structure internally stable. The diffractometer provides three degrees of motion – two for the sample stage (sample incidence angle θ and sample tilt χ) and one for the detector circle (scattering angle 2θ). The sample stage is placed on top of the diffractometer and it provides another four degrees of motion for the sample (the three translations *tx*, *ty*, *tz* and the polar angle ϕ). Therefore, the diffractometer and the sample stage provide in total six degrees of motion for the sample. Fig. 10 ▸(*a*) shows how the degrees of motion for the sample are staggered. Placing the translation stages above the diffractometer ensures that the rotation axis for θ and χ is always fixed and aligned with respect to 2θ. The polar angle ϕ is placed on top of the translation stages in order to keep the dimensions of the sample stage small. The movement range is listed in Table 4 ▸ (see also the supporting information). All motors of the diffractometer and sample stage are in-vacuum stepper motors. The sample receiver is thermally disconnected from the sample stage and connected to a liquid-flow helium cryostat through Cu braids, mounted on top of the XRD chamber. In order to keep the braids short while still allowing the full movement range of the the sample, the cryostat is placed on a manipulator that can follow the motion of the sample stage. In this configuration, temperatures below 20 K can be easily obtained at the sample receiver (16 K when the temperature at the cryostat is at 5 K).

The detector circle allows mounting multiple photodiodes and APDs that are used for diagnostics and X-ray detection. Their location can be adopted to the experimental needs (in steps of 10°). The detector circle also holds an ‘antenna’ (SMA plug connected to a coax cable) that is used for timing diagnostics. The breadboard provides the possibility of mounting additional equipment inside the XRD chamber. In particular, it will be used to support a laser-incoupling mirror for experiments requiring short focal length for the pump–probe laser (*e.g.* mid-IR and THz wavelengths). For laser wavelengths where this is not required, the laser-incoupling unit (LIN) of the SCS instrument is used (nearly collinear incoupling geometry with 2 m focal length for the XRD setup).

## 5. First high-resolution RIXS spectra of hRIXS

### 5.1. Settings and methods

The hRIXS spectrometer was commissioned together with the CHEM endstation and its solid- and liquid-sample environment. To benchmark the performance, static RIXS data were taken from well studied sample systems. Measurements from solid samples were performed at room temperature and a base pressure of 10^−7^ mbar. We selected NiO and La_2_CuO_4_. The data demonstrate the high-resolution and high-throughput capabilities of the hRIXS instrumentation at SCS. Optimized spectrometer settings can be kept in the same position for weeks, without degradation of resolution, as was observed during operation. In order to demonstrate the feasibility of liquid-jet studies at hRIXS, we measured liquid-water RIXS from a 40 µm cylindrical jet at a base pressure of 10^−3^ mbar.

The available polarization of the XFEL beam was linear horizontal, *i.e.* lying in the scattering plane (π polarization). All data were measured in the high-energy-resolution mode, using HRG in the spectrometer and the 150 lines mm^−1^ grating in the monochromator. For X-ray absorption (XAS) measurements, required to determine the excitation energy for RIXS measurements, we used a microchannel-plate detector placed at 85° scattering angle and operated to collect total fluorescence yield from the sample. The hRIXS spectrometer was placed at a scattering angle of 2Θ = 125°. The RIXS signal was collected using a (time-integrating) PI-MTE3 2048 × 2048 CCD from Teledyne Princeton Instruments (pixel size of 15 µm), mounted at γ = 25° incidence angle (standard detector hRIXS geometry). The cooling temperature was set to −50°C, the analog gain to high and digitization rate to 1 MHz. The exposure time was several seconds (depending on photon energy and flux), such that single photon counting was possible. An optical filter with a membrane was installed between the CHEM vessel and hRIXS grating chamber, in order to block optical light and protect the hRIXS vacuum from a liquid-jet environment: 200 nm Al film on 100 nm ParyleneC (from Luxel). Normalization of the signal intensity was done using an X-ray gas monitor (XGM) detector, located inside the SCS instrument hutch before the Kirkpatrick–Baez refocusing optics (Maltezopoulos *et al.*, 2019*a* ▸; Maltezopoulos *et al.*, 2019*b* ▸). The XGM delivers pulse-resolved signal utilized for fast data (here XAS) (Le Guyader *et al.*, 2023 ▸), as well as averaged signal that can be utilized for slow data (RIXS).

In order to initially find signal in the hRIXS spectrometer we used the diffraction peak from a multilayer sample. The final spectrometer alignment was performed on the specular reflection from a polished single-crystal sample (either NiO or CoO), which delivered the strongest non-resonant elastic signal with π-polarized X-rays.

The measured RIXS spectrum appears as lines on the 2D detector. Due to non-corrected aberrations, those lines are not straight, but slightly parabolic, depending on the settings of the spectrometer. For each setting, we calibrate the aberrations by fitting a parabola to the most intense line (usually the elastic line). Using this calibration we apply two methods to extract the actual spectrum, depending on the intensity of the signal: (i) if the signal is weak, such that individual photons can be seen, we fit those photons to a Gaussian peak, which reveals the position of the photon even more accurately than one pixel (Amorese *et al.*, 2019 ▸; Kummer *et al.*, 2017 ▸); (ii) for strong signals we employ a simple integration strategy – for each pixel we calculate its central energy following the aberration correction, then summing the intensity of pixels into one-pixel-wide bins, where the intensity is split between the two closest bins, proportionate to how close the central energy is to either bin.

Data analysis is done on-line, *i.e.* after each image taken the analysis is automatically performed. Data are stored in the XFEL data acquisition system, and can later be analysed off-line. We have developed a toolbox (Mercadier *et al.*, 2022 ▸) containing the relevant analysis procedures. It is based on the Python *xarray* package (Hoyer & Hamman, 2017 ▸) and is usually used within *Jupyter Notebooks* (Kluyver *et al.*, 2016 ▸).

### 5.2. Experimental data from solid samples

**Figure 11:**
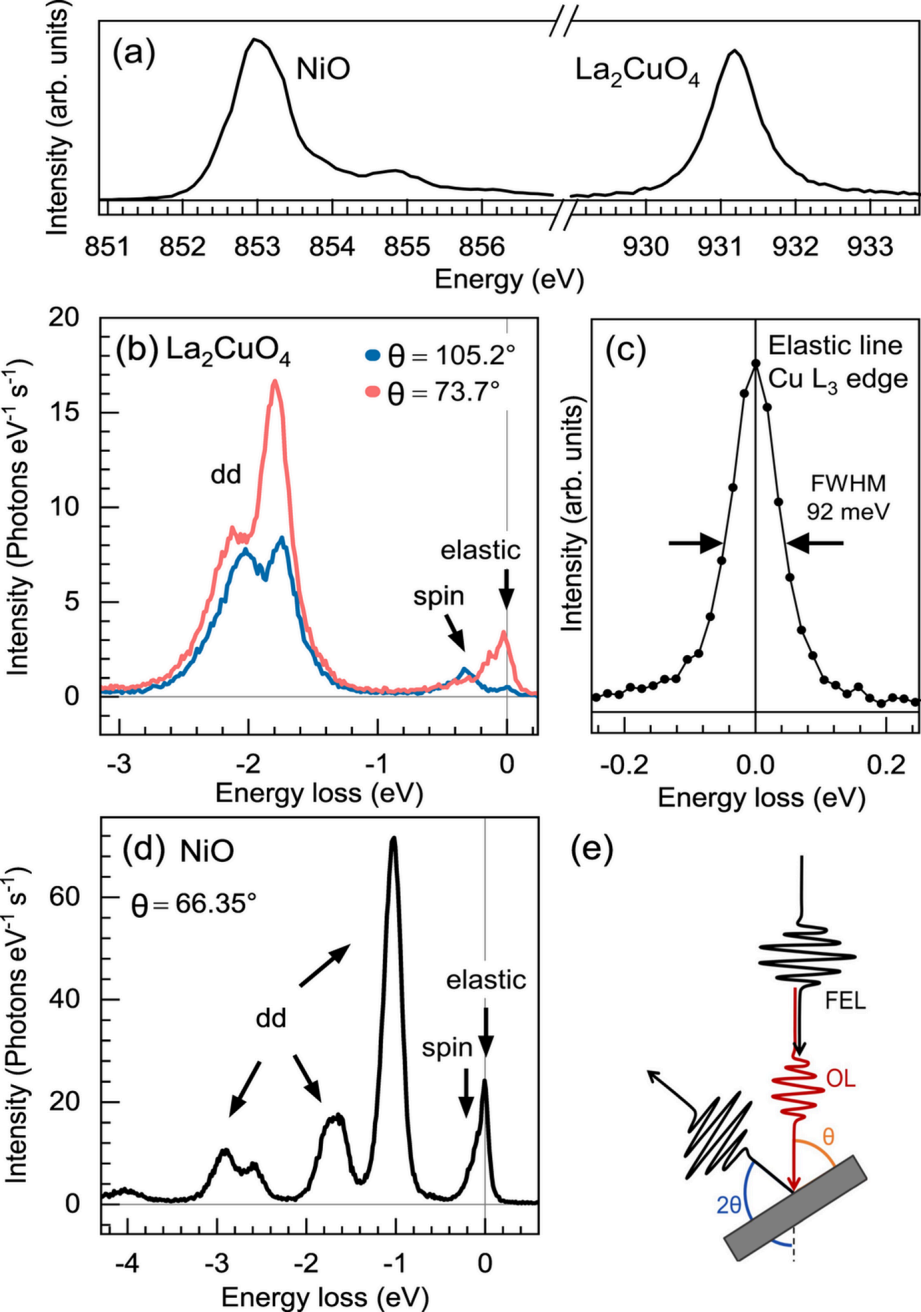
(*a*) XAS spectra of NiO and La_2_CuO_4_. (*b*) RIXS spectra of thin-film La_2_CuO_4_ at the Cu *L*_3_ edge measured at two different incident angles. The surface normal of the sample is parallel to the crystallographic *c*-direction. (*c*) Elastic line near specular conditions to estimate the combined resolution taken on La_2_CuO_4_. (*d*) RIXS spectrum of bulk-crystal NiO at the Ni *L*_3_ edge. The surface normal is the crystallographic *a*-direction. The elastic, magnetic and *dd* excitations are indicated in the spectra. (*e*) Schematic of the experimental geometry.

Fig. 11 ▸ shows Cu *L*_3_- (Ni *L*_3_-) edge spectra of a ∼48 nm-thick La_2_CuO_4_ film grown on LaSrAlO_4_ (single crystal NiO). The samples were at room temperature and the measurements were carried out at an intra-train repetition rate of 113 kHz and an attenuation factor of the X-ray beam of 30% that gives ∼0.22 µJ pulse^−1^ at the Cu *L*_3_ edge and ∼0.17 µJ pulse^−1^ at the Ni *L*_3_ edge as measured by the X-ray gas monitor (Maltezopoulos *et al.*, 2019*a* ▸). The focus on the sample was ∼8 µm  ×  200 µm (v × h). Under these conditions the samples did not show evident alteration due to radiation damage, as could be judged from the absorption and RIXS spectral shape. The XAS data shown in Fig. 11 ▸(*a*) were obtained by simultaneously scanning the undulator gaps and the monochromator incidence angle, a procedure used routinely at the SCS instrument (Le Guyader *et al.*, 2023 ▸; Lojewski *et al.*, 2023 ▸). The measured total energy resolution of the RIXS spectra in Figs. 11 ▸(*b*) and 11(*d*) (being a convolution of the SASE3 monochromator energy resolution and the hRIXS spectrometer energy resolution) was 93 meV FWHM at the Cu *L*_3_-edge [see Fig. 11 ▸(*c*)] and 80 meV FWHM at the Ni *L*_3_ edge. The detected count rate is comparable with that of equivalent static RIXS spectra measured at synchrotron-light-source beamlines (Braicovich *et al.*, 2010*a* ▸; Betto *et al.*, 2021 ▸; Ghiringhelli *et al.*, 2009 ▸; Betto *et al.*, 2017 ▸). The European XFEL repetition rate is compatible with an ∼100 kHz pump laser repetition rate, and can be increased by a factor of ten (see the supporting information for NiO data measured at 1.13 MHz repetition rate). The limiting factor would be the pump laser pulse energy at high repetition rates (particularly when using the OPA) and the sample damaging when increasing the average power irradiated through both the XFEL and the pump laser. The spectra correspond to different scattering geometries, resulting in different projections of the electrical-field vectors for the incident and exit X-ray beams on the crystallographic axes, as well as different momentum transfer along the Cu–O plane. As for La_2_CuO_4_, at θ ≃ 74° the in-plane component for the *dd* excitation peaks is more pronounced and the spin excitations have single magnon and bi-magnon contributions at comparable intensity. For θ ≃ 105°, where the momentum transfer is larger, the single magnon is better resolved, while its scattering cross section is bigger than that of the bi-magnon (Ament *et al.*, 2009 ▸; Braicovich*et al.*, 2010*b* ▸; Bisogni *et al.*, 2012 ▸). As for NiO [Fig. 11 ▸(*d*)], the *dd* features are all well resolved, while the spin excitations are at the limit of the experimental resolution. These spectra show that RIXS measured on prototypical strongly correlated materials and excited with ultra-short and intense XFEL pulses preserves their shape and can be interpreted in the same way as those measured at synchrotrons.

### 5.3. Experimental data from liquid samples

**Figure 12:**
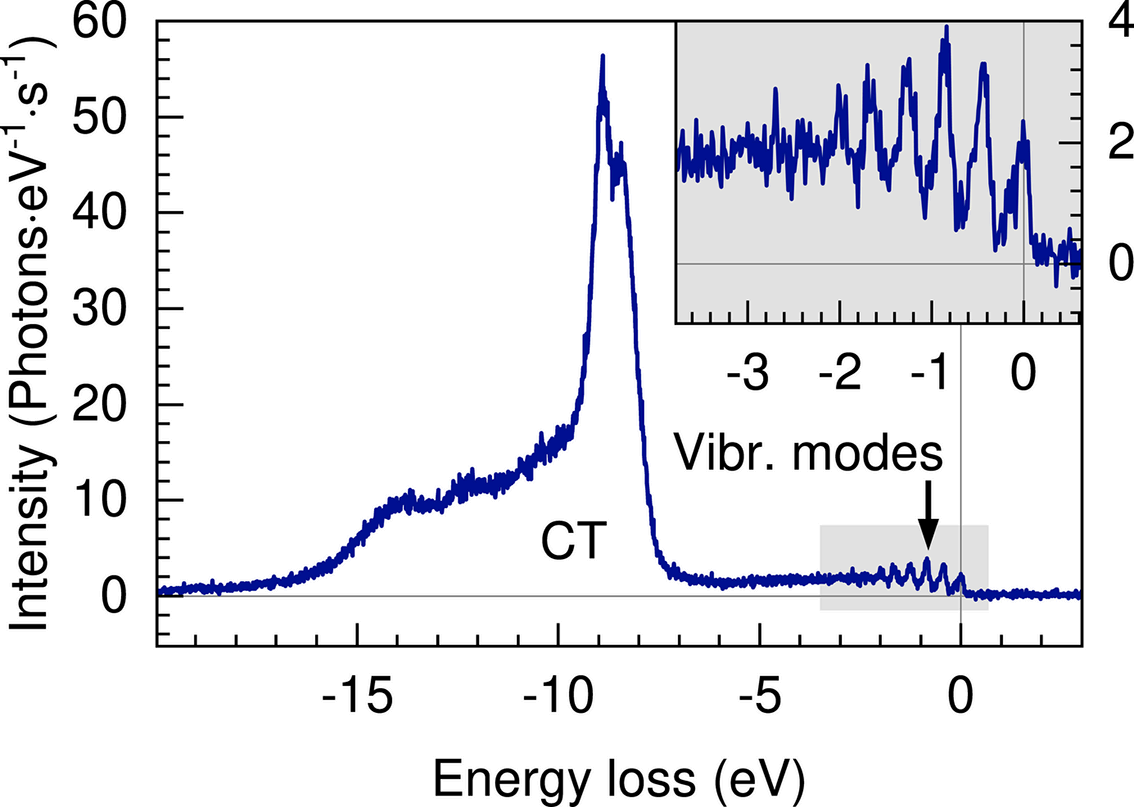
RIXS spectrum of liquid water obtained during commissioning at room temperature with the 1000 lines mm^−1^ grating. The inset shows a close-up of the vibrational progression.

The spectrum of liquid water from a liquid jet measured at 535 eV corresponding to the maximum of the O *K*-edge pre-edge feature is shown in Fig. 12 ▸. The spectrum exhibits the electronic excitations previously reported between 8 and 18 eV energy loss for liquid-cell measurements (Weinhardt *et al.*, 2012 ▸; Harada *et al.*, 2013 ▸; Vaz da Cruz *et al.*, 2019 ▸; Pietzsch *et al.*, 2022 ▸) and liquid-jet (Yin *et al.*, 2015 ▸; Lange *et al.*, 2010 ▸). The inset shows a close-up of the vibrational progression. This spectrum was measured at 1.13 MHz repetition rate, 450 pulses train^−1^, 100% transmission and 100 µm beamline exit slit (FEL output power of 500 µW). The polarization of the X-rays was linear horizontal (leading to a reduced cross section for vibrational excitations, compared with linear vertical polarization) and the spectrometer was placed at 2Θ = 125°. The diameter of the liquid jet was 40 µm and the flow rate was set to 1.2 ml min^−1^. The resulting spectrum was constructed from 20 min of acquisition. The pressure in the experimental chamber was at ∼4.5 × 10^−3^ mbar.

### 5.4. Performance of hRIXS at SCS

Table 5 ▸ displays an overview of the measured performance. A combined resolving power above 10000 is reached in the photon energy range 500–1000 eV. The values demonstrate that the energy resolving power of HTG and of the current high-resolution monochromator grating match very well (each around the design value of 10000 at 1000 eV, resulting in a combined resolving power of about 7000) (Gerasimova *et al.*, 2022 ▸), while the resolving power of HRG is significantly higher (design value a factor of three better). Depending on experimental requirements, there are three configurations that can be currently selected, optimizing either for energy resolution or throughput. When studying transition-metal monoxides (*e.g.* NiO) at the *L*_2,3_ edges, at 1.1 MHz repetiton rate high-resolution RIXS can be obtained within minutes using HRG. When working with HTG, the data output increases by about a factor of four to five (see also Fig. 2 ▸). For experiments where a modest resolving power of 3500 is sufficient, the flux can be potentially increased by another factor of six when using the high-throughput monochromator grating (Gerasimova *et al.*, 2022 ▸). This is particularly interesting for liquid-jet experiments, where sample concentrations are low. At the same time, when using the high-throughput monochromator grating the FEL pulse stretching is reduced, from about 80 fs pulse length (FWHM) at the Cu *L*_3_-edge (150 fs at the O *K*-edge) to about 30 fs (50 fs), improving the temporal resolution of the experiment. It should be mentioned that, when working with the high-resolution monochromator grating, the increased FEL pulse length will be the dominating contribution to the overall time resolution at SASE3, as the other contributions are lower (Schulz *et al.*, 2019 ▸; Schulz *et al.*, 2015 ▸; Rivas *et al.*, 2022 ▸; Grychtol *et al.*, 2021 ▸; Czwalinna *et al.*, 2021 ▸; Kirkwood *et al.*, 2019 ▸).

The present combined energy resolution of RIXS data is already sufficient to study crystal-field and spin excitations in cuprates and in other 3*d* transition metal oxides. However, at the moment the combined resolving power is limited by the beamline monochromator. When the high-resolution grating of the beamline monochromator is installed, the combined bandwidth will drop to ∼45 meV at the Cu *L*_3_-edge and comparatively even better at lower energies, where the edges of Ni, Co, Fe, Mn, Cr, V and Ti can be found. This means that not only orbital, charge-transfer and spin excitations but also phonons and charge excitations (Rossi *et al.*, 2019 ▸; Arpaia *et al.*, 2019 ▸) can be studied in a pump–probe fashion, exploiting the high-resolution RIXS at SCS.

The RIXS spectrum of water presented above as well as preliminary data on solutes demonstrate excellent agreement between data collected with hRIXS and data from synchrotron sources. This suggests that X-ray damage-free spectra are obtained using the liquid-jet sample delivery. This is aided by a rapid sample refresh due the small vertical beam size (<10 µm) and high jet velocity, which ensures fresh sample is introduced for every X-ray shot even at the MHz repetition rate within the EuXFEL burst. Further, it suggests that the high X-ray intensity does not induce detectable damage to the sample within the pulse length. Such conditions have a major impact on the variety of applications accessible because the full MHz repetition rate of EuXFEL can be employed, which typically gives between 4000 and 5000 X-ray pulses s^−1^. Previous RIXS experiments on 3*d* metal complexes at 120 Hz required high sample concentrations (≥300 m*M*), severely limiting which samples could be studied. Because the RIXS signal level and hence feasibility is proportional to both the sample concentration and solvent penetration depth, each sample system must be individually considered. That being said, with hRIXS at SCS we have found that experiments with a 50–100 m*M* concentration of a metal-organic complex are routine. Further, experiments down to 1 m*M* are likely feasible in high-penetration-depth solvents (alkanes, alcohols, *etc*.) for the late transition metal ions (Co and above). This fact combined with sample recirculation allows studies on the photochemical dynamics of a much broader range of systems than was previously accessible.

## 6. Summary and outlook

The combination of the high-repetition-rate European XFEL with the brilliance-driven photon-hungry technique of RIXS turns out to be a perfect match as seen from the swift data acquisition in the combined high energy resolution and femtosecond time information. Thus, excited-state detection and ultrafast dynamics are now routinely accessible with temporal resolution between 30 fs and 100 fs and an energy resolution of some tens of meV. In consequence, the full potential of dynamic RIXS is pushed towards the fundamental information content given by the transform limit in energy and time. This performance is achieved through the optical design of a VLS spherical-grating geometry, which has been optimized to balance energy range and resolving power, as well as throughput. In particular, an energy range from 500 eV to 1000 eV with a maximum resolution of 50 meV at the oxygen *K*-edge has been achieved. In the future an additional spectrometer grating can be mounted for further optimized performance on specific spectral regions or extending the photon energy range into the tender X-rays. The strengths and uniqueness of the chosen optical and mechanical design of the hRIXS spectrometer is that total resolution is currently limited by the energy resolution of the SASE3 monochromator grating and the pixel size of the X-ray detectors. We have intentionally built into the optical and mechanical design the ability to harvest future improved performance parameters on the soft X-ray SASE3 beamline and detector technologies. Foreseeable are the developments of large high-resolution beamline gratings, detectors with smaller pixel size and advanced timing modes. In particular, differential measurements between ultrafast stimulus (*i.e.* laser) ‘on’ versus ‘off’ at a rate within the European XFEL bunch trains will minimize the averaging influence of inter- and intra-bunch train jitter and further improve the statistics. Here, the rapid evolution of pixelated and fast-readout detectors is going to benefit hRIXS. In consequence, enhanced pump on–off contrast at highest duty cycle opens the path towards dilute active sites and potentially novel excitations with lower cross sections, such as dressed states of the ground state and pump-induced transient states. The modular design of the sample environments in combination with the confocal set-up of beamline focus and hRIXS spectrometer source point allows optimized sample environments to be exchanged without losing the alignment of the infrastructure. Currently, a dedicated UHV solid-state sample environment and liquid-phase chemistry environment are in operation. Moreover, potentially other complementary environments can be envisaged in the future. The availability of variable linear and circular polarization through the SASE3 Apple-X afterburner at the soft-X-ray branch of the European XFEL is going to give additional contrast mechanisms such as RIXS-XMCD (X-ray magnetic circular dichroism), where the dynamic response of magnetically ordered and chiral materials and molecules is going to benefit.

## Supplementary Material

Sections S1 to S3, including Figures S1 to S9 and Table S1. DOI: 10.1107/S1600577524010890/yi5154sup1.pdf

## Figures and Tables

**Table 1:** The main parameters for the high-resolution grating (HRG) and the high-transmission grating (HTG) of the hRIXS spectrometer

	HRG	HTG
*E*_0_ (mm)	900	600
*R* (mm)	64647	70114
*a*_0_ (mm^−1^)	3000	1000
*a*_1_ (mm^−2^)	1.232	0.446
*a*_2_ (mm^−3^)	5.35 × 10^−4^	2.06 × 10^−4^
*a*_3_ (mm^−4^)	3.5 × 10^−7^	1.6 × 10^−7^
Actual *s*′ (µrad r.m.s.)	0.18	0.11
Area (mm)	200 × 40	200 × 40
*c*/*d*	0.60	0.65
*h* (nm)	5	9
Micro-roughness (nm)	0.2	0.5
Coating thickness (nm)	29	40

**Table 2:** hRIXS spectrometer motion ranges [for stability, specified (measured) values are shown]

	Range	Stability
Grating pitch, α (grx)	83–92°	<0.09 (0.06) µrad
Grating horizontal motion, *r*_1_ (gtx)	900–2500 mm	<50 µm
Detector horizontal motion (dtx)	3500–50000 mm	<50 (0.03) µm
Detector vertical motion (dty)	1410–2210 mm	<3 µm
Scattering angle, 2Θ (rry)	65–145°	

**Table 3:** Motion ranges for CHEM sample environment

Motion	Range	Accuracy	Description
θ	±180°	0.01°	Sample incidence angle
*tx*	±7.5 mm	1 µm	Translation perpendicular to FEL
*tz*	±7.5 mm	1 µm	Translation along FEL
*ty*	100 mm		Vertical translation

**Table 4:** Motion ranges for XRD inner mechanics: detector circle (D), sample stage (S) and triple-rotating flange (TRF)

Motion	Range	Repeatability	Description
2θ	±180°	<0.002°	D scattering angle
θ	±180°	<0.002°	S incidence angle
χ	±30°	<0.002°	S tilt
ϕ	±90°	<0.1°	S polar angle
*tx*	±5 mm	<5 µm	S translation perpendicular to FEL
*tz*	±5 mm	<5 µm	S translation along FEL
*ty*	±5 mm	<5 µm	S vertical translation
2Θ	60–147°		TRF scattering angle

**Table 5:** Achieved performance when using the current high-resolution monochromator gratings hRIXS angular acceptance of about 47 mrad^2^ (with horizontal detector size of 30.7 mm). Photon flux at 1.1 MHz repetition rate and 400 pulses train^−1^ with 100% transmission, 50 µm beamline exit slit and spot size of 12 µm × 100 µm (v × h). Machine at 11.5 GeV. When using the high-transmission monochromator grating the photon flux on the sample increases by about a factor of six, while the resolving power decreases to about 3500.

Photon energy (eV)	Photon flux at sample (photons s^−1^)	hRIXS grating	Combined resolving power
850	1.0 × 10^13^	HRG (3000 lines mm^−1^)	10600
930	1.3 × 10^13^	HRG (3000 lines mm^−1^)	10100
530	1.6 × 10^12^	HTG (1000 lines mm^−1^)	10600
850	As above	HTG (1000 lines mm^−1^)	7080
