# Supplementary material for: The Heisenberg-RIXS instrument at the European XFEL
Source: J Synchrotron Radiat. 2025 Jan 1;32(Pt 1):29–45. doi: 10.1107/S1600577524010890 (PMC11708868; doi:10.1107/S1600577524010890)
Supplement: Supplementary file 1 [file s-32-00029-sup1.pdf]

Supporting Information for article:

The Heisenberg-RIXS instrument at the European XFEL

Justine Schlappa,[a,*] Giacomo Ghiringhelli,[b,c,#] Benjamin E. Van Kuiken,[a] Martin Teichmann,[a] Piter S. Miedema,[a] Jan Torben Delitz,[a] Natalia Gerasimova,[a] Serguei Molodtsov,[a,d,e] Luigi Adriano,[a] Bernard Baranasic,[a] Carsten Broers,[a] Robert Carley,[a] Patrick Gessler,[a] Nahid Ghodrati,[a] David Hickin,[a] Le Phuong Hoang,[a] Manuel Izquierdo,[a] Laurent Mercadier,[a] Giuseppe Mercurio,[a] Sergii Parchenko,[a] Marijan Stupar,[a] Zhong Yin,[a,f] Leonardo Martinelli,[b,g] Giacomo Merzoni,[a,b] Ying Ying Peng,[b,h] Torben Reuss,[i] Sreeju Sreekantan Nair Lalithambika,[i,j] Simone Techert,[i,j,†] Tim Laarmann,[i,k,◦] Simo Huotari,[l] Christian Schroeter,[m] Burkhard Langer,[m] Tatjana Giessel,[m] Jana Buchheim,[n] Grzegorz Gwalt,[n] Andrey Sokolov,[n] Frank Siewert,[n] Robby Buechner,[o] Vinicius Vaz da Cruz,[o] Sebastian Eckert,[o] Chun-Yu Liu,[o,p] Christian Sohrt,[o] Christian Weniger,[o] Annette Pietzsch,[o] Stefan Neppl,[p,q] Friedmar Senf,[p] Andreas Scherz[a,%] and Alexander Föhlisch[o,p,$]

[a]European XFEL, Holzkoppel 4, Schenefeld, 22869, Germany, [b]Dipartimento di Fisica, Politecnico di Milano, piazza Leonardo da Vinci 32, I-20133 Milano, Italy, [c]CNR-SPIN, Dipartimento di Fisica, Politecnico di Milano, I-20133 Milano, Italy, [d]Institute of Experimental Physics, TU Bergakademie Freiberg, Leipziger Str. 23, 09599 Freiberg, Germany, [e]Center for Efficient High Temperature Processes and Materials Conversion (ZeHS), TU Bergakademie Freiberg, Winklerstrasse 5, 09599 Freiberg, Germany, [f]Present address: International Center for Synchrotron Radiation Innovation Smart, Tohoku University, Sendai 980-8572, Japan, [g]Present address: Physik-Institut, University of Zürich, Winterthurerstrasse 190, CH-8057 Zürich, Switzerland, [h]Present address: International Center for Quantum Materials, School of Physics, Peking University, Beijing 100871, China, [i]Deutsches Elektronen-Synchrotron DESY, Notkestraße 85, 22607 Hamburg, Germany, [j]Institute of X-ray Physics, Goettingen University, Friedrich Hund Platz 1, 37077 Goettingen, Germany, [k]The Hamburg Centre for Ultrafast Imaging CUI, Luruper Chaussee 149, 22761 Hamburg, Germany, [l]Department of Physics, University of Helsinki, P.O. Box 64, FI-00014 Helsinki, Finland, [m]BESTEC GmbH, Am Studio 2b, 12489 Berlin, Germany, [n]Department Optics and Beamlines, Helmholtz Zentrum Berlin für Materialien und Energie GmbH, Albert-Einstein-Strasse 15, 12489 Berlin, Germany, [o]Institute Methods and Instrumentation for Synchrotron Radiation Research, Helmholtz-Zentrum Berlin für Materialien und Energie GmbH, Albert-Einstein-Straße 15, 12489 Berlin, Germany, [p]University of Potsdam, Institute of Physics and Astronomy, Karl-Liebknecht-Straße 24/25, 14476 Potsdam, Germany, [q]Present address: Paul Scherrer Institut, Forschungsstrasse 111, 5232 Villigen PSI, Switzerland.

[*] justine.schlappa@xfel.eu, [#]giacomo.ghiringhelli@polimi.it, [%]andreas.scherz@xfel.eu, [†]simone.techert@desy.de, [◦]tim.laarmann@desy.de, and [$]alexander.foehlisch@helmholtz-berlin.de

S1. Optical design detailed description

S1.0. Introduction

Following the procedure used to design the SAXES spectrometer for the Swiss Light Source (Ghiringhelli et al, 2006, *Rev. Scientific Instrum.* **77** 113108), the VLS grating parameters can be calculated analytically starting from few external parameters following a univocal optimization algorithm. The input parameters are the total length of the instrument (L_0), the central groove density (a_0), the spot size on the sample (S_1), the detector spatial resolution (S_1) and the angle of incidence on the detector (γ). $L_0$is dictated by the floor space available. a_0 is chosen in consideration of the photon energy range and determines the grating dispersion properties, ie the resolution of the spectrometer. The "central" photon energy (E_0) for the optimization and the corresponding angle of incidence α_0 must also be chosen, the latter in consideration of grating efficiency calculated separately. The main optimization criterion is that the contribution to the instrumental line width (LW) of the spectrometer coming from S_1 and S_2 have to be equal, to minimize their quadratic sum. Once the grating VLS parameters (radius of curvature R, linear a_1 and quadratic a_2 terms of the polynomial expansion $a(w) = a_0 + a_1 w + a_2 w^2 + \cdots$ are thus determined, it is possible to calculate analytically the working positions of the spectrometer (α, β, r_1, r_2) for any given energy (E). As there is no unique (α,r_1) combination that can fulfill both the focus condition and the coma aberration cancellation, one can choose to fix either of them and calculate the other parameter. The choice of the (α,r_1) combination can be dictated by efficiency considerations or mechanical limitations of the spectrometer.

Below we provide all equations needed to perform these calculations.

S1.1. Definitions

**Input Parameters**	**Units**	**Description**
E_0	eV	Reference energy for optimization
$L_0 = r_{1,0} + r_{2,0}$	mm	Total length at the reference energy
a_0	mm^{-1}	Central groove density
α_0	deg	Incidence angle on grating at the reference energy
γ_0	deg	Incidence angle on the detector (from surface) at E_0
S_1	μm	Spot size on the sample = Source size (FWHM)
S_2	μm	Detector spatial resolution in the dispersion direction
s'	μrad	Grating surface slop error, FWHM
$k = +1$		Diffraction order, positive if internal ($\beta < \alpha$)
K = 0.001239842	mm eV	Wavelength – Energy conversion for photons
**Calculated parameters**		
β_0	deg	Diffraction angle from grating at the reference energy
$r_{1,0}$	mm	Entrance arm length at the reference energy
$r_{2,0}$	mm	Exit arm length at the reference energy
R	mm	Radius of curvature (tangential)
a_1	mm^{-2}	VLS linear term
a_2	mm^{-3}	VLS quadratic term
**Variables**		
E	eV	Photon energy
α	deg	Incidence angle on grating, at generic energy
β	deg	Diffraction angle from grating, at generic energy
r_1	mm	Entrance arm length, at generic energy
r_2	mm	Exit arm length, at generic energy

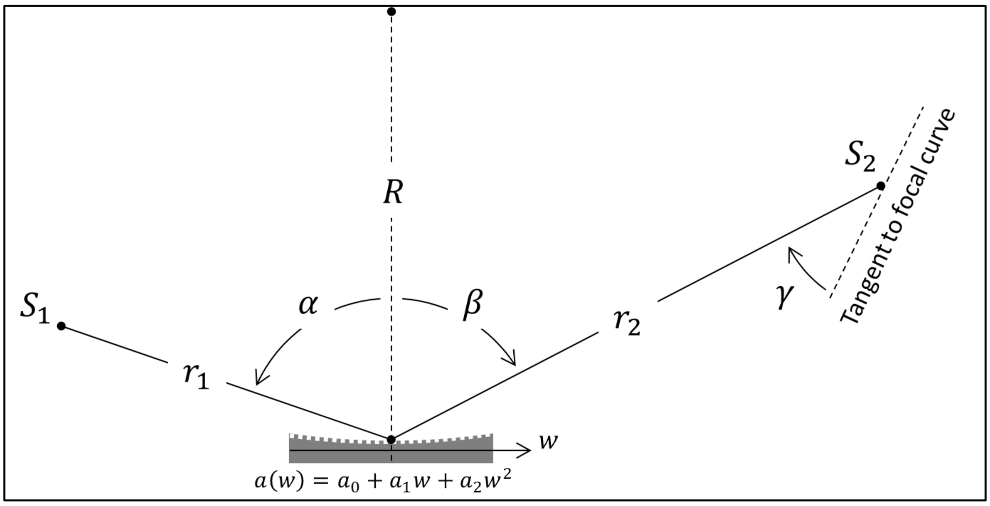

Fig. S1. Optical scheme for a spherical grating VLS spectrometer and definition of the geometrical parameters.

S1.2. Basic equations

From the Berkeley's orange book (https://cxro.lbl.gov/x-ray-data-booklet) and (Osborn & Callcott, 1995, *Rev. Scientific Instrum.* **66,** 3131), we can get the following system of equations. See subsection S1.5 for more details.

$$\sin\alpha - \sin\beta = \frac{kK}{E} a_0 \qquad \text{grating diffraction} \qquad \text{(ES1)}$$

$$\frac{\cos^2\alpha}{r_1} + \frac{\cos^2\beta}{r_2} = \frac{\cos\alpha + \cos\beta}{R} + \frac{kK}{E} a_1 \qquad \text{focus} \qquad \text{(ES2)}$$

$$\tan\gamma = \frac{\cos\beta}{2\sin\beta - r_2\left(\frac{\tan\beta}{R} + \frac{a_1}{a_0}\right)} \qquad \text{focal line grazing angle} \qquad \text{(ES3)}$$

$$\frac{\sin\alpha}{2r_1}\left(\frac{\cos^2\alpha}{r_1} - \frac{\cos\alpha}{R}\right) - \frac{\sin\beta}{2r_2}\left(\frac{\cos^2\beta}{r_2} - \frac{\cos\beta}{R}\right) + \frac{1}{3}\frac{kK}{E} a_2 = 0 \qquad \text{coma correction} \qquad \text{(ES4)}$$

$$\Delta E_1 = S_1 \frac{\cos\alpha}{r_1 a_0 kK} E^2 \qquad \text{source contribution to LW} \qquad \text{(ES5)}$$

$$\Delta E_2 = S_2 \sin\gamma \frac{\cos\beta}{r_2 a_0 kK} E^2 \qquad \text{detector contribution to LW} \qquad \text{(ES6)}$$

$$\Delta E_{\mathrm{SE}} = \frac{2s\prime}{\tan(\alpha - \beta)} E \qquad \text{slope-error contribution to LW} \qquad \text{(ES7)}$$

S1.3. *Optimization of the grating parameters*

As mentioned in the introduction, the independent input parameters are: L_0 (nominal length of the spectrometer, dictated by mechanical considerations and floor space availability), a_0 (density of the grating lines at the center), E_0 (reference energy, usually in the middle of the working range), α_0 (chosen to optimize the grating efficiency at E_0), S_1 (FWHM of the x-ray spot on the sample in the dispersion direction of the spectrometer grating), S_2 (actual spatial resolution of the detector in the

detection surface along the dispersion direction), γ (the average working angle of incidence of photons onto the detector, measured from the surface). From the equation (ES1) we get

$$\beta_0 = \mathrm{asin}\left(\sin\alpha_0 - \frac{kK}{E_0}a_0\right). \tag{ES8}$$

We then equalize the two contributions to the spectrometer line width (ES4, ES5), and get the entrance and exit arms at the reference energy:

$$r_{1,0} = \frac{S_1\cos\alpha_0}{S_1\cos\alpha_0+S_2\sin\gamma\cos\beta_0}L_0 \tag{ES9}$$

$$r_{2,0} = L_0 - r_{1,0}. \tag{ES10}$$

We impose can now combine (ES2) and (ES3) to obtain the values of the radius of curvature R and of the linear VLS parameter a_1:

$$R = \frac{\frac{E_0}{kK}(\cos\alpha_0+\cos\beta_0)-a_0\tan\beta_0}{\frac{E_0}{kK}\left(\frac{\cos^2\alpha_0}{r_{1,0}}+\frac{\cos^2\beta_0}{r_{2,0}}\right)-\frac{a_0}{r_{2,0}}\left(2\sin\beta_0-\frac{\cos\beta_0}{\tan\gamma_0}\right)} \tag{ES11}$$

$$a_1 = \frac{E_0}{kK}\left(\frac{\cos^2\alpha_0}{r_{1,0}}+\frac{\cos^2\beta_0}{r_{2,0}}-\frac{\cos\alpha_0+\cos\beta_0}{R}\right). \tag{ES12}$$

We can finally calculate the quadratic VLS parameter a_2:

$$\begin{aligned} a_2 &= -3\frac{E_0}{kK}\left[\frac{\sin\alpha_0}{2r_{1,0}}\left(\frac{\cos^2\alpha_0}{r_{1,0}}-\frac{\cos\alpha_0}{R}\right)-\frac{\sin\beta}{2r_{2,0}}\left(\frac{\cos^2\beta_0}{r_{2,0}}-\frac{\cos\beta_0}{R}\right)\right] = \\ &= \frac{3}{4}\frac{E_0}{kK}\left[\frac{\sin 2\alpha_0}{r_{1,0}}\left(\frac{1}{R}-\frac{\cos\alpha_0}{r_{1,0}}\right)-\frac{\sin 2\beta_0}{r_{2,0}}\left(\frac{1}{R}-\frac{\cos\beta_0}{r_{2,0}}\right)\right]. \end{aligned} \tag{ES13}$$

S1.4. Determination of working points for generic energy

Once the grating specified, for any given energy E, we want to determine the values of α, β, r_1 and $r_2$that fulfill the focusing conditions and the cancellation of the coma aberration. We have 4 parameters and 3 equations (ES1,ES2,ES4) relating them. We choose to fix α and calculate the other three parameters as function of it.

We know that Eq (ES1) can provide β for any given α and E:

$$\beta = \mathrm{asin}\left(\sin\alpha - \frac{kK}{E}a_0\right), \tag{ES14}$$

so below we consider that β is known, without expressing it explicitly as in Eq. (ES14). We are thus left with 2 equations and 3 variables, α, r_1 and r_2. We first rewrite (ES2) and (ES4) for convenience:

$$\frac{\cos\beta}{r_2} = \frac{1}{\cos\beta}\left(\frac{\cos\alpha+\cos\beta}{R}+\frac{kK}{E}a_1-\frac{\cos^2\alpha}{r_1}\right) \tag{ES15}$$

$$\frac{\sin\alpha}{2}\left(\frac{\cos\alpha}{r_1}-\frac{1}{R}\right)\frac{\cos\alpha}{r_1}-\frac{\sin\beta}{2}\left(\frac{\cos\beta}{r_2}-\frac{1}{R}\right)\frac{\cos\beta}{r_2}+\frac{1}{3}\frac{kK}{E}a_2 = 0\ . \tag{ES16}$$

Then, if we define a parameter A (remember that α and β have been fixed) and a new variable $x(r_1)$:

$$A = \left(\frac{\cos\alpha+\cos\beta}{R}+\frac{kK}{E}a_1\right)\frac{1}{\cos\beta} \tag{ES17}$$

$$x = \frac{\cos\alpha}{r_1}, \tag{ES18}$$

we can rewrite (ES15) and (ES16) in a more compact way

$$\frac{\cos\beta}{r_2} = A - \frac{\cos\alpha}{\cos\beta}x \tag{ES19}$$

$$\frac{\sin\alpha}{2}\left(x - \frac{1}{R}\right)x - \frac{\sin\beta}{2}\left(\frac{\cos\beta}{r_2} - \frac{1}{R}\right)\frac{\cos\beta}{r_2} + \frac{1}{3}\frac{kK}{E}a_2 = 0\ . \tag{ES20}$$

By substituting $\frac{\cos\beta}{r_2}$ from (ES19) into (ES20) we get

$$\frac{\sin\alpha}{2}x^2 - \frac{\sin\alpha}{2R}x - \frac{\sin\beta}{2}\left[\left(A^2 - 2A\frac{\cos\alpha}{\cos\beta}x + \frac{\cos^2\alpha}{\cos^2\beta}x^2\right) - \frac{A}{R} + \frac{\cos\alpha}{\cos\beta}\frac{x}{R}\right] + \frac{1}{3}\frac{kK}{E}a_2 = 0 \tag{ES21}$$

that we can rearrange to obtain a second order equation in the variable x

$$c_1x^2 - c_2x + c_3 = 0 \tag{ES22}$$

with

$$c_1 = \sin\alpha - \frac{\cos^2\alpha}{\cos^2\beta}\sin\beta \tag{ES23}$$

$$c_2 = \frac{\sin\alpha}{R} - \left(2A - \frac{1}{R}\right)\frac{\cos\alpha}{\cos\beta}\sin\beta \tag{ES24}$$

$$c_3 = \frac{2}{3}\frac{kK}{E}a_2 + A\left(\frac{1}{R} - A\right)\sin\beta\ . \tag{ES25}$$

We can thus find the solutions

$$x = \frac{c_2 + \sqrt{c_2^2 - 4c_1c_3}}{2c_1} \tag{ES26}$$

$$r_1 = \frac{\cos\alpha}{x} \tag{ES27}$$

$$r_2 = \frac{\cos^2\beta}{\frac{\cos\alpha + \cos\beta}{R} + \frac{kK}{E}a_1 - \frac{\cos^2\alpha}{r_1}}\ . \tag{ES28}$$

We can now use (ES5,ES6,ES7) to calculate the total LW of the spectrometer in the working position, by a making quadratic summation:

$$\Delta E_{\text{spectro}} = \sqrt{(\Delta E_1)^2 + (\Delta E_2)^2 + (\Delta E_{\text{SE}})^2} \tag{ES29}$$

These formulas can be easily implemented in a worksheet or a code in any programming language. Usually the value of α is kept quite close to α_0, not to degrade the grating efficiency that is optimum at α_0 for a broad energy range once the grove profile parameters have been decided.

S1.5. Some remarks on equations

Equations (ES1) and (ES2) are very well known and widely used. They can be derived from equations in section 4.3 of Berkeley's orange book (https://cxro.lbl.gov/x-ray-data-booklet). Equation (ES4) can also be derived from there. However, it is important to note that it is different from equation (8b) in (Osborn & Callcott, 1995, *Rev. Scientific Instrum.* **66,** 3131), which reads $\frac{\sin\alpha}{r_1}\left(\frac{\cos^2\alpha}{r_1} - \frac{\cos\alpha}{2R}\right) - \frac{\sin\beta}{r_2}\left(\frac{\cos^2\beta}{r_2} - \frac{\cos\beta}{2R}\right) + \frac{kK}{E}a_2 = 0$. This discrepancy has generated some confusion so that in

(Ghiringhelli et al, 2006, *Rev. Scientific Instrum.* **77** 113108) still another version was used. Equation (ES4) is definitely the correct one, giving results consistent with ray tracing simulations.

Equation (ES3) is taken from (Osborn & Callcott, 1995, *Rev. Scientific Instrum.* **66,** 3131). For convenience, we derive it here again.

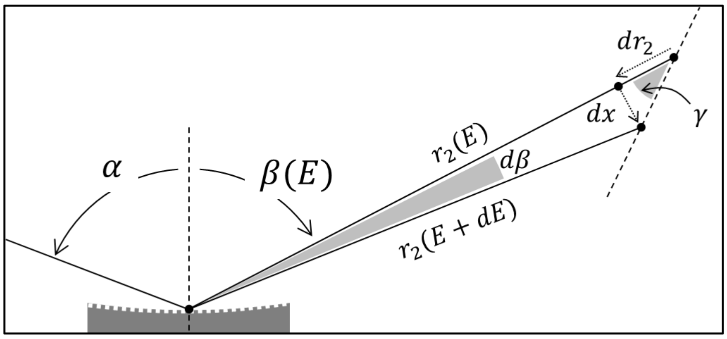

Fig. S2. Geometrical parameter definition used to derive equation (ES3).

From the figure S2, with the convention that γ is positive, and noting that β increases and r_2 decreases when E increases, we can write

$$\tan\gamma = \frac{dx}{-dr_2} = \frac{r_2 d\beta}{-dr_2} = -r_2\frac{d\beta}{dr_2} \tag{ES30}$$

$$\frac{1}{\tan\gamma} = -\frac{1}{r_2}\frac{dr_2}{d\beta}. \tag{ES31}$$

From equation (ES2), we write $r_2(\beta)$ in a simplified form

$$r_2(\beta) = \frac{F_1(\beta)}{F_0+F_2(\beta)}, \tag{ES32}$$

where

$$F_0 = \frac{\cos\alpha}{R} - \frac{\cos^2\alpha}{r_1} + \frac{a_1}{a_0}\sin\alpha \tag{ES33}$$

$$F_1(\beta) = \cos^2\beta \tag{ES34}$$

$$F_2(\beta) = \frac{\cos\beta}{R} - \frac{a_1}{a_0}\sin\beta. \tag{ES35}$$

We need the derivatives of $F_{1,2}$

$$F_1' = -2\sin\beta\cos\beta \tag{ES36}$$

$$F_2' = -\frac{\sin\beta}{R} - \frac{a_1}{a_0}\cos\beta \tag{ES37}$$

to write

$$\frac{1}{r_2}\frac{dr_2}{d\beta} = \frac{F_0+F_2}{F_1}\left[\frac{F_1'}{F_0+F_2} - \frac{F_1F_2'}{(F_0+F_2)^2}\right] = \frac{F_1'}{F_1} - \frac{F_2'}{F_0+F_2}\,. \tag{ES38}$$

Therefore, after substituting the expressions we get

$$-\frac{1}{\tan\gamma} = -2\tan\beta + \frac{\frac{\sin\beta}{R}+\frac{a_1}{a_0}\cos\beta}{\frac{\cos\alpha+\cos\beta}{R}-\frac{\cos^2\alpha}{r_1}+\frac{a_1}{a_0}(\sin\alpha-\sin\beta)}\,. \tag{ES39}$$

Using equations (ES1,ES2) we see that

$$\frac{\cos^2\beta}{r_2} = \frac{\cos\alpha+\cos\beta}{R} - \frac{\cos^2\alpha}{r_1} + \frac{a_1}{a_0}(\sin\alpha - \sin\beta) \tag{ES40}$$

so we can simplify equation (ES39)

$$-\frac{1}{\tan\gamma} = -2\tan\beta + \left(\frac{\sin\beta}{R} + \frac{a_1}{a_0}\cos\beta\right)\frac{r_2}{\cos^2\beta} \tag{ES41}$$

that can be finally rewritten as in equation (ES3)

$$\tan\gamma = \frac{\cos\beta}{2\sin\beta - r_2\left(\frac{\tan\beta}{R}+\frac{a_1}{a_0}\right)}. \tag{ES42}$$

S1.6. Ray tracing

a) b)

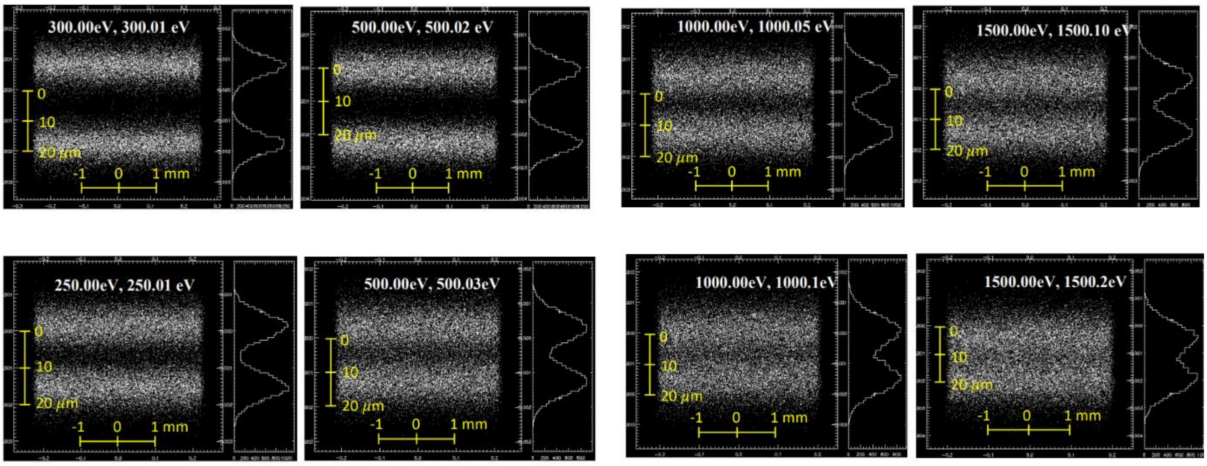

Fig. S3: Ray-tracing simulations for hRIXS spectrometer for photon energies of 250 eV - 500 eV (a) and 1,000 eV -1,500 eV (b) made with Shadows. Top figures show results for HRG (1,000 l/mm grating) and bottom figures for HTG (3,000 l/mm), respectively. The source has Gaussian spatial distribution with FWHM S_1 = 5 μm and angular divergence of 1 mrad in both directions. The spatial intensity distribution on the surface of the detector of two monochromatic lines of energy indicating at the top of each panel are shown.

S2. Mechanical design, specifications and performances

a)

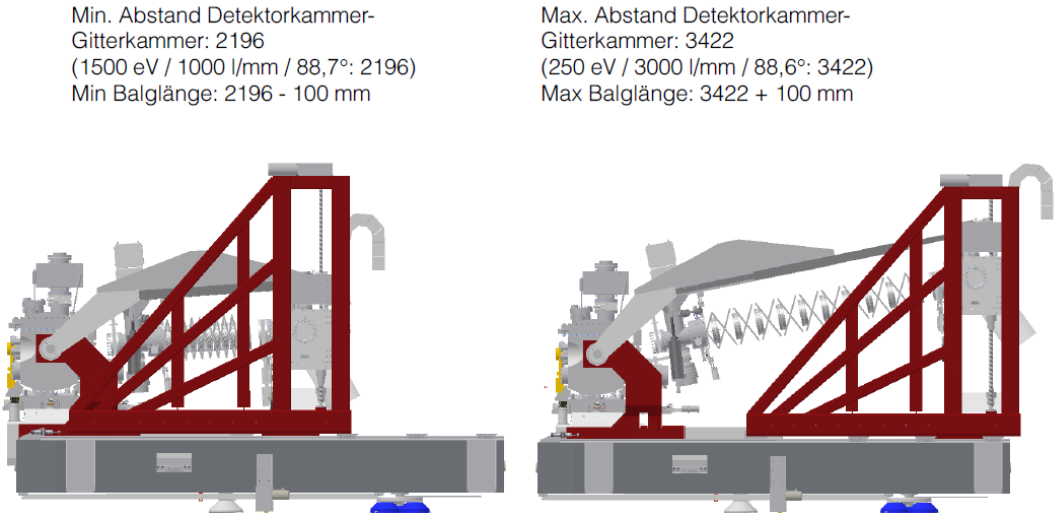

b)

3000L/mm		no access possible				best resolution				
r1 (entrance arm)		r1 < 1300 mm				best transmission				

	88°	88,1°	88,2°	88,3°	88,4°	88,5°	88,6°	88,7°	88,8°	88,9°	89°
250 eV							1636	1505	1377	1251	1128
300 eV						1609	1486	1365	1248	1132	1019
400 eV				1716	1598	1481	1368	1256	1148	1041	936
500 eV			1779	1661	1546	1434	1323	1216	1110	1007	905
600 eV		1870	1752	1636	1523	1412	1304	1198	1094	992	
700 eV		1857	1740	1625	1513	1403	1295	1190	1086	985	
800 eV		1852	1736	1621	1509	1400	1292	1187	1084	983	
900 eV	1970	1851	1735	1621	1509	1400	1292	1187	1084	983	
1000 eV	1971	1853	1737	1623	1511	1401	1294	1188	1085	984	
1100 eV	1975	1856	1740	1626	1514	1404	1296	1191	1088	986	
1200 eV	1979	1860	1744	1630	1518	1408	1300	1194	1090	989	
1300 eV	1983	1865	1748	1634	1522	1412	1304	1198	1094	992	
1400 eV	1988	1870	1753	1639	1526	1416	1308	1201	1097	995	
1500 eV	1993	1875	1758	1643	1531	1420	1312	1205	1101	998	

1000L/mm
r1 (entrance arm)

	88°	88,1°	88,2°	88,3°	88,4°	88,5°	88,6°	88,7°	88,8°	88,9°	89°
250 eV			1925	1794	1666	1541	1420	1301	1186	1073	963
300 eV		2046	1913	1783	1656	1533	1412	1294	1179	1067	958
400 eV		2044	1912	1783	1657	1534	1413	1296	1181	1068	959
500 eV		2054	1923	1793	1667	1543	1423	1304	1189	1076	
600 eV	2202	2067	1935	1806	1679	1555	1434	1315	1199	1085	
700 eV	2215	2081	1949	1819	1692	1567	1445	1326	1209		
800 eV	2227	2093	1961	1831	1704	1579	1456	1336	1218		
900 eV	2239	2105	1973	1843	1715	1590	1466	1346	1227		
1000 eV	2250	2116	1984	1854	1726	1600	1476	1355	1236		
1100 eV	2260	2126	1994	1863	1735	1609	1485	1363	1244		
1200 eV	2269	2135	2003	1872	1744	1618	1493	1371			
1300 eV	2277	2143	2011	1881	1752	1626	1501	1378			
1400 eV	2285	2151	2019	1889	1760	1633	1508	1385			
1500 eV	2292	2158	2026	1896	1767	1640	1515	1392			

Fig. S4: Horizontal movement range for the hRIXS detector chamber (a) and grating chamber (b). The movement range gives a large working range for each photon energy, so that optimization either on energy resolution or on transmission is possible. The demands on the mechanical construction are high, e.g. the length of bellow connecting the grating and detector chamber can change from 2096 mm to 3522 mm (a).

a)

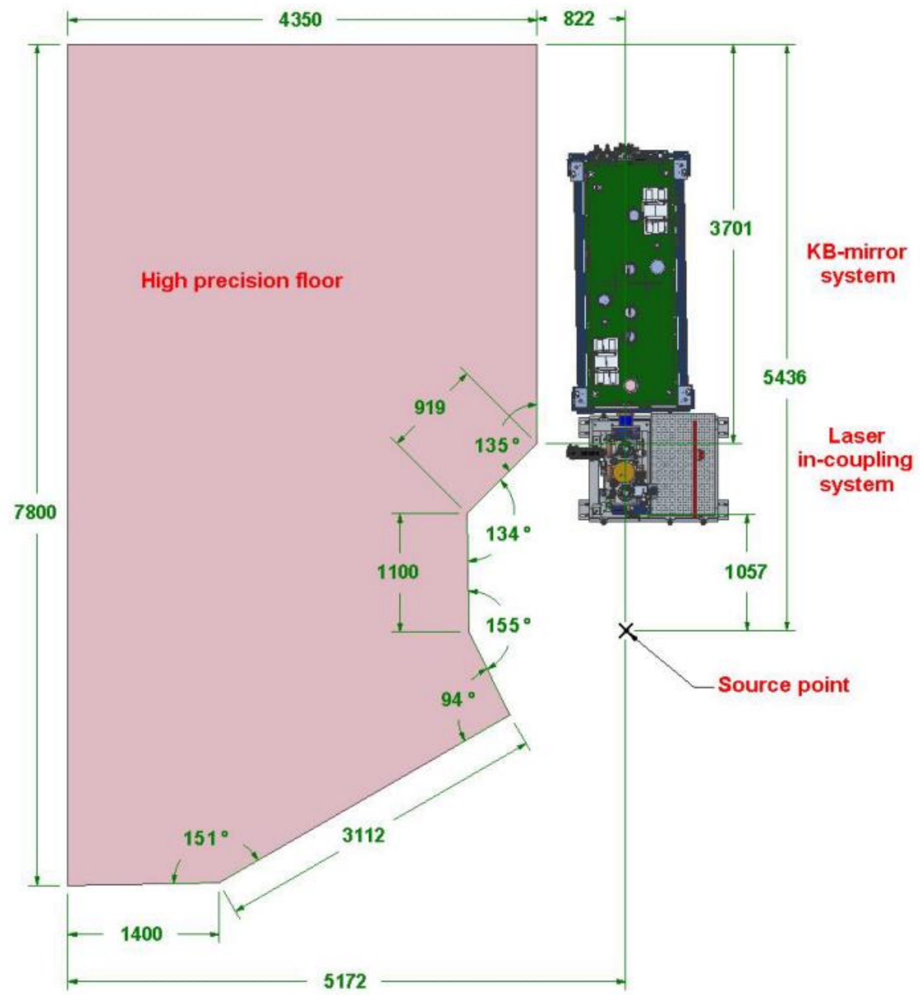

b)

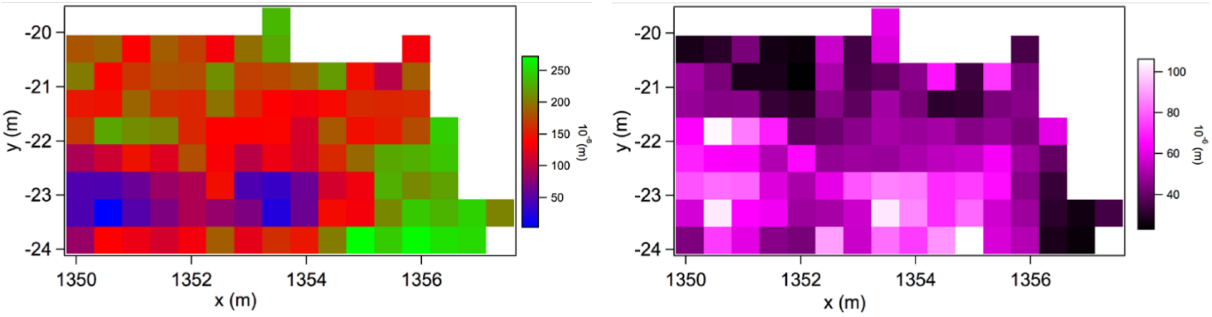

Fig. S5: Dimensions and location of the high-quality floor (HQF) inside the SCS hutch (a). The length along the beamline direction is about 7 m and perpendicular to the beamline around 4 m. The shape of the floor had to be adopted to the specific situation inside SCS hutch and leave enough space (next to the source point) for installation of rails for hRIXS rotation. Geodetic measurement results for height variation of the HQF (left) and planarity averaged over 1 m (right) (b). Results are shown in µm, the horizontal (vertical) axis refers to coordinates inside SCS hutch along (perpendicular to) the beamline. The maximum height variation over the entire HQF area is in the rage of 250 µm.

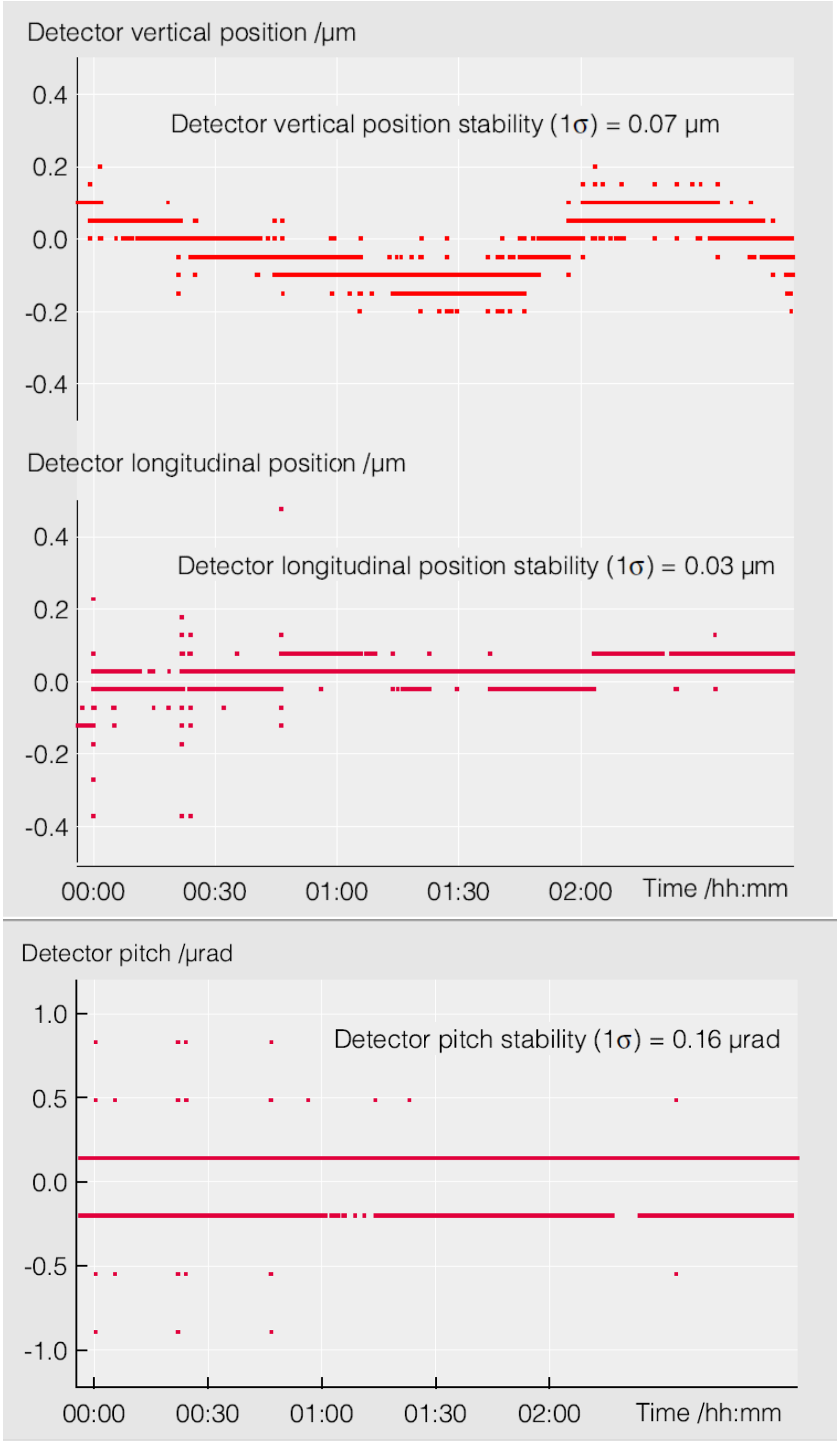

Fig. S6: Stability measurements for detector-chamber position after installation of the hRIXS spectrometer at the source point. Displayed is encoder data for detector height, distance and pitch, measured over duration of 3 h (the data is neither averaged or filtered).

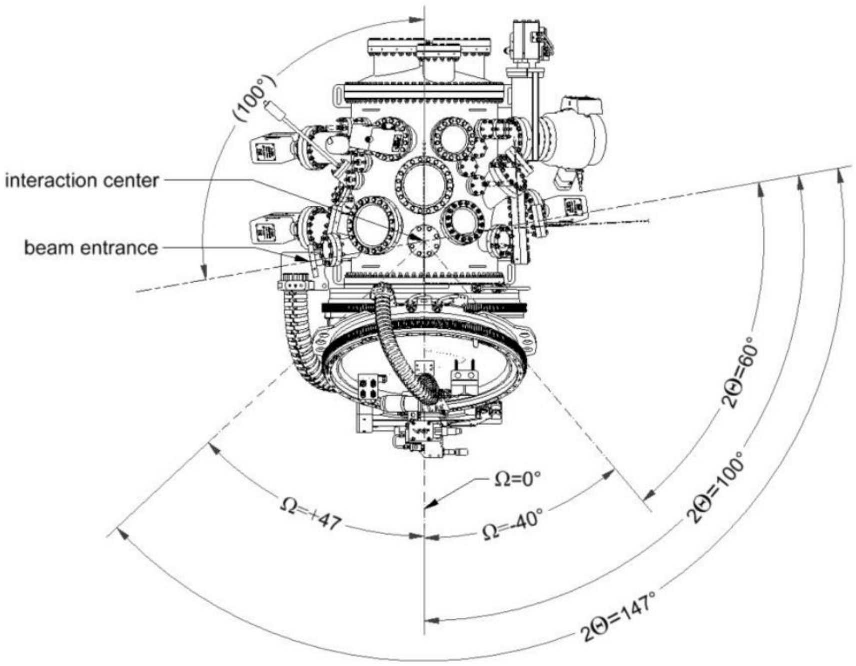

Fig. S7: Swivel range of the triple-rotating flange (TRF) of the XRD chamber. The vessel is oriented with an offset of 10° in respect to the FEL beam, in order to maximize the accessible TRF back-scattering angle (Two-theta of 147°). The minimum TRF forward-scattering angle is 60° in Two-Theta.

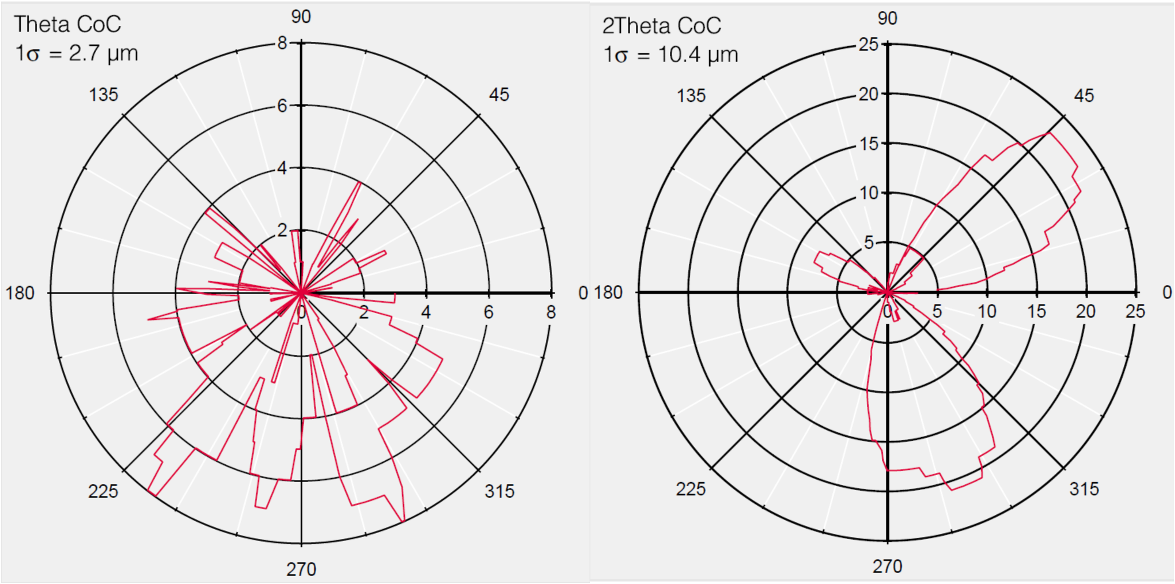

Fig. S8: Circle-of-confusion data (dial gauge signal) for sample Theta and detector Two-Theta rotation of the in-vacuum XRD diffractometer.

S3. Additional static RIXS spectra

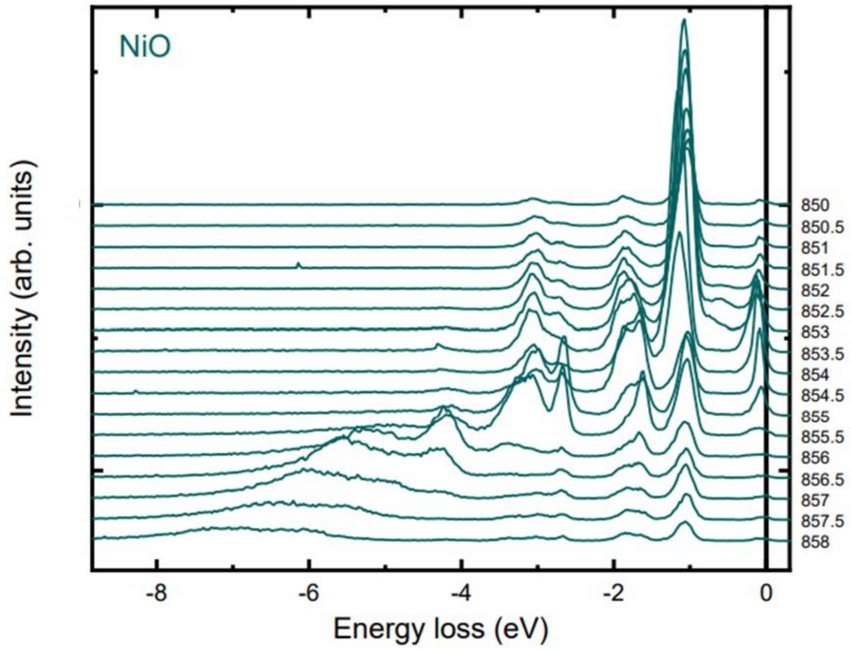

Fig. S9: RIXS spectra obtained from single-crystal NiO at room temperature across the Ni L_3 resonance. Acquisition time per spectrum was 60 s. Data was taken at 1.1 MHz repetition rate, 400 pulses per train and 100% transmission.
